# Prodrug‐Loaded Zirconium Carbide Nanosheets as a Novel Biophotonic Nanoplatform for Effective Treatment of Cancer

**DOI:** 10.1002/advs.202001191

**Published:** 2020-11-05

**Authors:** Quan Liu, Zhongjian Xie, Meng Qiu, Inseob Shim, Yunlong Yang, Sisi Xie, Qinhe Yang, Dou Wang, Shiyou Chen, Taojian Fan, Bo Ding, Ziheng Guo, Dickson Adah, Xinhuang Yao, Yuhua Zhang, Hong Wu, Zongze Wu, Chaoying Wei, Hongzhong Wang, Hyeong Seok Kim, Qingshuang Zou, Qiaoting Yan, Zhen Cai, Jong Seung Kim, Li‐Ping Liu, Han Zhang, Yihai Cao

**Affiliations:** ^1^ Department of Hepatobiliary and Pancreas Surgery The 2nd Clinical Medical College (Shenzhen People's Hospital) of Jinan University Shenzhen 518020 P. R. China; ^2^ Integrated Chinese and Western Medicine Postdoctoral Research Station Jinan University Guangzhou Guangdong 510632 P. R. China; ^3^ Shenzhen Second People's Hospital The First Affiliated Hospital of Shenzhen University and Collaborative Innovation Center for Optoelectronic Science and Technology of Shenzhen University Shenzhen 518060 P. R. China; ^4^ Shenzhen International Institute for Biomedical Research Shenzhen 518116 P. R. China; ^5^ Key Laboratory of Marine Chemistry Theory and Technology (Ocean University of China) Ministry of Education Qingdao 266100 P. R. China; ^6^ Department of Chemistry Korea University Seoul 02841 Korea; ^7^ Department of Microbiology Tumor and Cell Biology Karolinska Institute Stockholm 17177 Sweden; ^8^ Department of Cellular and Genetic Medicine School of Basic Medical Sciences Fudan University Shanghai 200032 P. R. China; ^9^ School of Traditional Chinese Medicine Jinan University Guangzhou Guangdong 510632 P. R. China; ^10^ Department of Respiratory Disease The Fourth Hospital of Jinan Jinan Shandong 250031 P. R. China; ^11^ Department of Pancreatic Surgery West China School of Medicine Sichuan University Chengdu 610041 P. R. China; ^12^ State Key Laboratory of Respiratory Disease Department of Infection and Immunity Guangzhou Institutes of Biomedicine and Health Chinese Academy of Sciences Guangzhou Guangdong 510530 P. R. China; ^13^ University of Chinese Academy of Sciences Beijing 100049 P. R. China; ^14^ Department of clinical laboratory Shenzhen Sun Yat‐sen Cardiovascular Hospital Shenzhen Guangdong 518020 P. R. China; ^15^ Shenzhen Public Service Platform on Tumor Precision Medicine and Molecular Diagnosis Shenzhen Guangdong 518020 P. R. China

**Keywords:** angiogenesis, chemotherapy, photothermal therapy, prodrug, zirconium carbide

## Abstract

Conventional chemotherapy and photothermal therapy (PTT) face many major challenges, including systemic toxicity, low bioavailability, ineffective tissue penetration, chemotherapy/hyperthermia‐induced inflammation, and tumor angiogenesis. A versatile nanomedicine offers an exciting opportunity to circumvent the abovementioned limitations for their successful translation into clinical practice. Here, a promising biophotonic nanoplatform is developed based on the zirconium carbide (ZrC) nanosheet as a deep PTT‐photosensitizer and on‐demand designed anticancer prodrug SN38‐Nif, which is released and activated by photothermia and tumor‐overexpressed esterase. In vitro and in vivo experimental evidence shows the potent anticancer effects of the integrated ZrC@prodrug biophotonic nanoplatform by specifically targeting malignant cells, chemotherapy/hyperthermia‐induced tumor inflammation, and angiogenesis. In mouse models, the ZrC@prodrug system markedly inhibits tumor recurrence, metastasis, inflammation and angiogenesis. The findings unravel a promising biophotonic strategy for precision treatment of cancer.

## Graphical abstract

A biophotonic nanoplatform based on the zirconium carbide (ZrC) nanosheet as a deep photothermal therapy‐photosensitizer and on‐demand designed anticancer prodrug SN38‐Nif is developed. Both physically controlled and bioresponsive mode are employed to smartly release drugs. The ZrC@prodrug system markedly inhibits tumor recurrence, metastasis, inflammation, and angiogenesis. The findings unravel a promising biophotonic strategy for precision treatment of cancer.

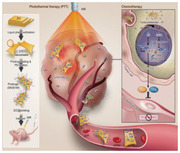

Cancer is one of the leading causes of death in both developed and developing countries.^[^ ^1^ ^]^ Surgery, chemotherapy, and radiation therapy are gold‐standard therapeutic modalities for various cancers. However, the available statistics indicate that these modalities are ineffective in treating tumor recurrence and metastasis, resulting in poor patient survival. Phototherapy including photothermal therapy (PTT) is a new emerging field of cancer therapy that has attracted extensive research interests, owing to their non‐replaceable advantages: 1) Minimal harm to healthy normal tissues; 2) noninvasiveness, and 3) efficient anti‐cancer activity.^[^ ^2^ ^]^ However, conventional PTT strategies suffer from several main problems, including limited tissue penetration, treatment‐related complications, treatment‐augmented inflammation, and hyperthermia‐induced angiogenesis,^[^ ^3^ ^]^ which may lead to tumor recurrence and metastasis.^[^ ^4^ ^]^

The PTT‐based anti‐cancer strategy relies on various photosensitive nanoparticles to generate local hyperthermia for damaging cancer cells under near‐infrared radiation (NIR).^[^ ^5^, ^6^ ^]^ In order to increase NIR tissue penetration ability, a desirable approach is to develop additional PTT photosensitizers (PSs) within the second NIR (NIR‐II) biowindow. Additionally, a new concept of biophotonic nanoplatforms integrating phototherapy and photocontrolled chemotherapy has emerged for precision treatment of cancer.^[^ ^7^ ^]^ However, the lack of specificity of inherently toxic anticancer agents is one of the main obstacles of the currently available photo‐chemotherapeutic agents. Thus, various physical, chemical, and biological methods have been developed to improve physicochemical properties and to deliver drugs more precisely to tumor tissues, including an enzyme‐activated prodrug^[^ ^8^ ^]^ and a nanoparticle encapsulation‐based method.^[^ ^9^ ^]^ Importantly, over the course of the last decades, previous work from our laboratory and others has made significant progress through synthesizing a large variety of prodrugs to alter the cancer microenvironment by increasing levels of intracellular thiols,^[^ ^10^ ^]^ significantly decreasing extracellular pH levels,^[^ ^11^ ^]^ reducing hypoxia,^[^ ^12^ ^]^ elevating concentrations of extra‐ and intracellular reactive oxygen species (ROS),^[^ ^13^ ^]^ and high expression of enzymes (such as carboxylesterase, esterase, quinone oxidoreductase).^[^ ^14^ ^]^ Other studies showed that a bare‐prodrug is still cytotoxic. For example, irinotecan (Iri) efficiently kill malignant cells, but also undesirably affects non‐tumor cells, including blood cells, epithelial cells, and commensal bacteria in cancer patients. Consequently, Iri‐based therapy often accompanies with high toxicity profiles such as diarrhea, primarily neutropenia, and sporadically certain severe life‐threatening toxicities.^[^ ^15^ ^]^ An enzyme‐activated prodrug system in combination with nano‐technology may solve these problems. Particularly, the PTT‐prodrug system can achieve tumor specific targeting.

Inspired by the excellent PTT performance of transitional metal carbide Ti_3_C_2_,^[^ ^16^ ^]^ zirconium (Zr), a transition metal within the same group but in a larger period than Ti, is used to obtain better thermoplasmonic effects because of the external heavy atom effect.^[^ ^17^ ^]^ Moreover, one unique feature of Zr (IV) is its higher oxidation state compared with those of the metal elements in group I, II, and III. Owing to the high charge density and bond polarization, a strong affinity between Zr and carboxylate O atoms can occur, which may facilitate its drug loading. Therefore, the ZrC NSs exhibits an excellent NIR‐II photothermal performance with a photothermal conversion efficiency (PTCE) of 62.1% under 1064 nm irradiation and an ultrahigh drug loading capacity of ≈800%.^[^ ^18^ ^]^ In addition, unlike GO, TMDCs, BP, Ti_3_C_2_, Zr is widely distributed in biological systems under physiological conditions because of its rich biological activities.^[^ ^19^ ^]^ Its low toxicity further favors the development and application of Zr in the biomedical field.^[^ ^20^ ^]^

Conventional therapy may induce inflammation and angiogenesis, and metastasis via apoptotic tumor cell‐induced macrophage chemotaxis and proinflammatory cytokines.^[^ ^21^ ^]^ Angiogenesis is also a critical hallmark of malignant tumor growth.^[^ ^22^ ^]^ Recent studies suggested that enhanced signaling contributes to VEGF‐independent tumor angiogenesis through a COX‐2/prostaglandin E2 (PGE_2_) axis.^[^ ^23^ ^]^ COX‐2/PGE_2_ inhibition may potentiate VEGF therapies. Thus, COX‐2‐specific inhibitors, such as Nif, may be used to inhibit COX‐2‐associated signal pathways, impair tumor angiogenesis^[^ ^24^ ^]^ and a premetastatic and protumorigenic microenvironment with decreased proinflammatory cells^[^ ^25^ ^]^ in tumor tissues. Currently, developing a well‐designed prodrug based on SN38‐induced chemotherapy and Nif‐induced antiangiogenic therapy against tumor poses a great challenge to researchers, and the development of prodrugs in combination with PTT for improved cancer treatment has not been reported.

In this work, a promising ultrathin 2D zirconium carbide (ZrC) nanosheet (NSs) is explored for anticancer activity by combining both NIR‐II photothermal PS and photocontrolled drug delivery platform. The prodrug‐loaded ZrC (ZrC@prodrug) accumulates in tumor tissues by enhancing permeability and retention (EPR), followed by releasing prodrugs through ZrC‐generated photo‐hyperthermia. Carboxylesterase, a frequently overexpressed enzyme in cancer cells, but not healthy normal cells,^[^ ^26^ ^]^ mediates the conversion of the prodrug into Nif and SN38 (**Figure** **1**a). In addition to its high‐performance PTT, ZrC also allows selective accumulation of prodrugs and consequently improving the bioavailability of SN38. We show that the COX‐2 inhibitor Nif significantly inhibits tumor angiogenesis through the COX‐2/PGE_2_/VEGF pathway. ZrC@prodrug modulates the tumor environment (TME) and reduces chemotherapy/hyperthermia‐induced inflammation, which may collectively create a pro‐metastatic environment.^[^ ^27^ ^]^ Importantly, both ZrC and the prodrug exhibit negligible toxicity. Finally, highly effective in vivo tumor ablation was demonstrated in various tumor xenografts by combining photothermal therapy and chemotherapy. This new combination approach demonstrates that the ZrC@prodrug biophotonic nanoplatform effectively ablates tumor growth, recurrence, and metastasis.

**Figure 1:**
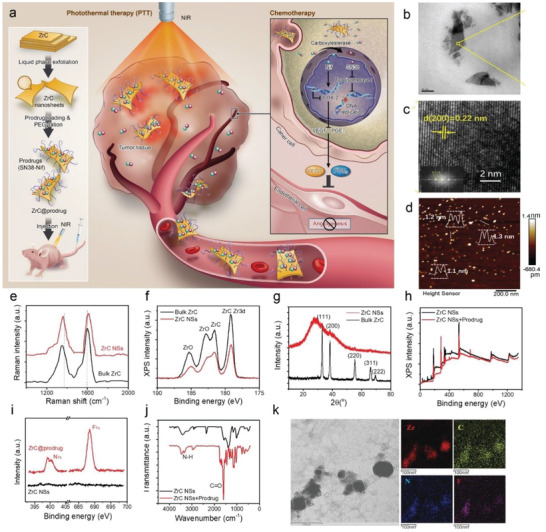
Characterizations of ZrC NSs and ZrC@prodrug for precise cancer treatment. a) Schematic illustration of 2D biodegradable ZrC@prodrug (SN38‐Nif) for in vivo tumor photo‐chemotherapy. Liquid‐phase exfoliation (LPE) of ZrC NSs in water. b) TEM and c) HRTEM images (inset shows the corresponding SAED pattern) of ZrC NSs. d) AFM image of ZrC NSs. e) Raman scattering spectra acquired from bulk ZrC and ZrC NSs, respectively. f) XPS spectra of bulk ZrC and ZrC NSs, respectively. g) XRD of bulk ZrC and ZrC NSs, respectively. h,i) XPS spectra of ZrC NSs and ZrC@prodrug. j) FTIR spectra of ZrC NSs. k) STEM images of ZrC@prodrug.

ZrC NSs were prepared by the liquid‐phase exfoliation method^[^ ^28^ ^]^ (Figure 1a). Energy dispersive X‐ray spectroscopy (EDS) spectrum was employed to determine the stoichiometric ratio of ZrC NSs. As shown in Figure S1 (Supporting Information), the stoichiometric formula of ZrC is Zr_0.93_C_1_O_0.19_. The morphology of the ZrC NSs was characterized by transmission electron microscopy (TEM) and atomic force microscopy (AFM). The TEM image (Figure 1b) shows the ultrathin structure of ZrC NSs and their typical lateral size is ≈50–200 nm, which is consistent with dynamic light scattering analysis (Figure S2c, Supporting Information). The crystallinity of the ZrC was studied by high‐resolution TEM (HRTEM) (Figure 1c) and selected‐area electron diffraction (SAED) (Figure 1c, inset). As shown in the HRTEM images, lattice fringes with an interlayer distance of 0.22 nm were observed and corresponded to the (200) planes of the ZrC crystal. The fast Fourier transform (FFT) of the crystal lattice showed the expected crystallographic lattice reflections of ZrC, which belongs to *Fm*3*m* space group. The AFM image (Figure 1d) provided further evidence for the ultrathin nanosheet morphology of ZrC that the thickness of the ZrC NSs falls within the range of 1.1–1.4 nm, which is consistent with the dimension of single or double layer ZrC NSs. Raman spectra of the bulk ZrC and ZrC NSs are shown in Figure 1e. The chemical composition of both bulk ZrC and exfoliated ZrC NSs was characterized using X‐ray photoelectron spectroscopy (XPS) (Figure 1f). The X‐ray diffraction (XRD) patterns of bulk ZrC and exfoliated ZrC NSs show similar reflection peaks (Figure 1g), which were found to come from the *fcc* structure of ZrC. All characterizations of the dimensions, composition and crystal features confirm the successful exfoliation of ZrC NSs.

XPS was further employed to examine the binding ability of prodrug to ZrC NSs (Figure 1h). A careful study in the magnified region (Figure 1i) verified the significant peaks at 399 and 688 eV corresponded to N1s and F1s after prodrug loading onto ZrC NSs compared to the as‐prepared ZrC NSs. Furthermore, the Fourier transform infrared (FTIR) spectrum (Figure 1j) indicated that the peaks at 1627 and 2920 cm^−1^ attributable to C=O and NH stretching vibrations belong to prodrug. In Figure 1k, scanning transmission electron microscopy (STEM) with energy dispersive X‐ray spectroscopy (EDS) mapping of ZrC@prodrug showed the co‐localization of Zr, C, F, and N elements. All of the above characterizations confirmed the successful prodrug loading onto ZrC NSs. The ZrC NSs and PEGylated ZrC NSs show excellent dispersion and stability in physiological medium and serum, due to the hydrophilic and negatively charged surface, as shown in Figures S2 and S3 (Supporting Information).

The exfoliated ZrC NSs were used in photothermal experiments. Strong absorption is a prerequisite for photothermal agents. In **Figure** **2**a, the ZrC NSs showed strong absorption in both NIR‐I and NIR‐II biowindows, and the extinction coefficient at 808 and 1064 nm were estimated to be 8.8 and 8.3 L g^−1^ cm^−1^, respectively (Figure 2b). The tissue penetration depth was also tested in both NIR‐I and NIR‐II biowindows. As shown in Figure S4 (Supporting Information), the power density residue of NIR‐II light is much higher than NIR‐I light, indicating a better tissue penetration depth of light in NIR‐II biowindow. The photothermal conversion efficiency (PTCE) is the most important property of a PS and determines its efficiency for PTT. The photothermal performance was investigated with ZrC suspensions exposed to an 808 or 1064 nm laser. As irradiation time increased, the temperature of ZrC suspensions under both wavelength irradiations increased from 25 °C to more than 60 °C after 10 min at a relatively low concentration (100 µg mL^−1^) (Figure 2c). Through the cooling period in one photothermal cycle (Figure 2d,e),^[^ ^29^ ^]^ the PTCE of the ZrC NSs at NIR‐I and NIR‐II windows were calculated to be 52.1% and 62.1%, respectively, indicating that the constructed ZrC NSs can efficiently convert NIR‐I and NIR‐II lights into heat. As shown in Figure 2f, this PTCE was significantly higher than those of other photothermal agents, such as Au nanoparticles (21%),^[^ ^30^ ^]^ black phosphorus quantum dots (BPQDs, 28.4%),^[^ ^6^ ^]^ antimonene quantum dots (AMQDs, 45.5%),^[^ ^31^ ^]^ and Ti_3_C_2_ NSs (30.6%).^[^ ^32^ ^]^ The excellent PTCE of ZrC is derived from the enhanced spin‐orbital coupling effect, due to the heavy atom Zr.^[^ ^17^ ^]^ Figure 2g shows six photothermal cycles for ZrC NSs suspension (25 µg mL^−1^) under 808 and 1064 nm irradiations, and the absorption spectra remain almost unchanged (Figure 2h), indicating the excellent photothermal stability of ZrC NSs.

**Figure 2:**
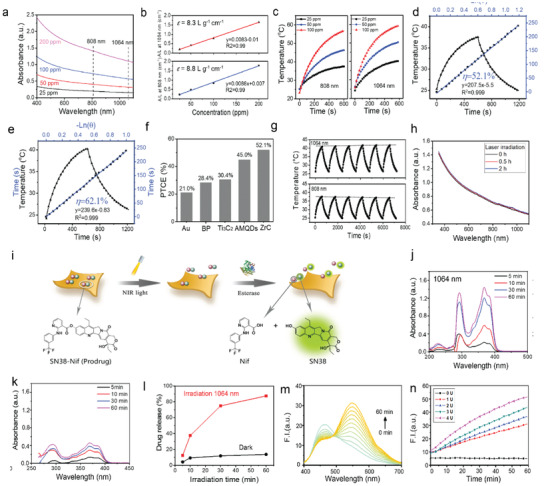
Photothermal performance of ZrC, and design strategy of prodrug and its activation behavior. a) UV–vis–NIR absorption spectra of ZrC NSs suspensions with various concentrations. b) Extinction coefficients of ZrC NSs suspensions under 808 and 1064 nm irradiation, respectively. c) Temperature change curves with different NSs suspensions concentrations under 808 and 1064 nm irradiation, respectively. d,e) Calculation of the photothermal‐conversion efficiency at 808 nm (NIR‐I) and 1064 nm (NIR‐II), respectively. f) Photothermal conversion efficient of Au NRs, BPQDs, Ti_3_C_2_ NSs and ZrC NSs. g) Heating curves of ZrC NSs suspensions in water for 6 laser on/off cycles (1.0 W cm^−2^) under the irradiation with a NIR‐I or NIR‐II laser, respectively. h) UV–vis–NIR absorption spectra of as‐prepared ZrC NSs suspensions and under irradiation for 0.5 and 2 h. i) Schematic illustration of the release and activation mechanism of prodrug‐loaded ZrC NSs, and the molecular structures of prodrug (SN38‐Nif), Irinotecan and Nif. Photocontrolled drug release profiles j) with and k) without light irradiation, and l) the absorption maximum peaks changes of prodrug. m) Fluorescence spectra changes of prodrug (10 × 10^−6^ m) in the presence of esterase (1 U mL^−1^). n) Fluorescence response of prodrug (10 × 10^−6^ m) as a function of esterase concentration.

The fabricated ZrC NSs could load the on‐demand designed prodrug with very high efficiency of (800% in weight, Figure S5, Supporting Information), due to the ultralarge specific surface of 2D layered structure and the high binding affinity between prodrug and ZrC NSs as confirmed by XPS in Figure 1h. The drug loading and activation mechanism of prodrug‐loaded ZrC NSs was schematically illustrated in Figure 2i. Once the prodrug is loaded on ZrC NSs, it could be released only under NIR irradiation. The photocontrolled prodrug release behavior was investigated through spectral changes under physiological conditions with UV–vis absorption spectra. As shown in Figure 2j–l and Figure S6 (Supporting Information) the prodrug can be released under both irradiation of 1064 and 808 nm. In Figure S6 (Supporting Information), the disappeared peak of N1s in XPS spectrum further verified the successful prodrug release after irradiation. Subsequently, the carboxylic ester bond of prodrug could react with esterase which is overexpressed in cancer cells, to generate fluorescent SN38 and Nif. The esterase‐triggered prodrug activation was investigated through FTIR (Figure S7, Supporting Information) and spectral changes under physiological conditions with fluorescent spectroscopy. As shown in Figure 2m,n, prodrug exhibited weak fluorescence at 460 nm without esterase, while the fluorescence at 560 nm increased dramatically when added with esterase, confirming that prodrug could be activated by esterase. Lastly, results showed that prodrugs in ZrC NSs could be activated and had a function against tumor cells at various concentrations (**Figure** **3**e,f). In the following sections, the 1064 nm laser was employed for biological experiments due to the better tissue penetration depth and higher PTCE compared with 808 nm laser.

**Figure 3:**
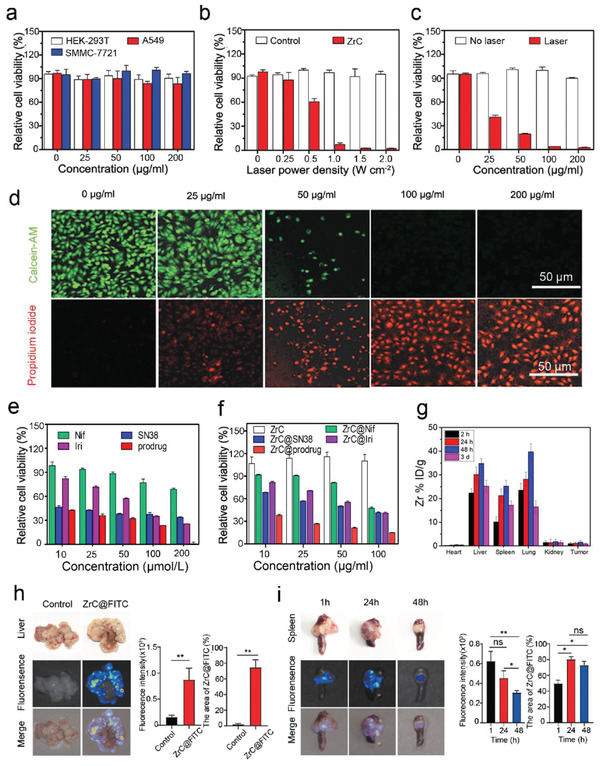
Biocompatibility, photothermal performance, cytotoxicity in vitro and biodistribution of ZrC nanoplatform with prodrug. a) Relative viabilities of HEK‐293T, A549, and SMMC‐7721 cells after incubation with ZrC NSs at various concentrations (0, 25, 50, 100, and 200 µg mL^−1^). b,c) Relative viabilities of SMMC‐7721 cells after ZrC NSs (100 µg mL^−1^)‐mediated photothermal ablation at different laser power densities (0, 0.25, 0.5, 1.0, 1.5, and 2 W cm^−2^) and various concentrations (0, 25, 50, 100, and 200 µg mL^−1^). Error bars were based on the standard deviations (SD) of four parallel samples. d) Fluorescence imaging of ZrC‐induced photothermal ablation of various concentrations (0, 25, 50, 100, and 200 µg mL^−1^) stained with Calcein‐AM (green, live cells) and propidium iodide (red, dead cells). Images share the same scale bar (50 µm). e) Relative cell viability of SMMC‐7721 cells treated with Nif, SN38, Iri or prodrug at various concentrations (10, 25, 50, 100, and 200 µmol L^−1^) for 48 h. f) Relative cell viability of SMMC‐7721 cells treated with ZrC, ZrC@Nif, ZrC@SN38, ZrC@Iri, or ZrC@prodrug at various concentrations (10, 25, 50, and 100 µg mL^−1^) after 48 h incubation. g) Biodistribution of Zr (% ID of Zr per gram of tissues) in main tissues and tumors at 2, 24, 48 h, and 3 days after the intravenous administration of ZrC dispersed in PBS. h) Biodistribution of ZrC@FITC in tumor and the adjacent liver normal tissues with a metastatic CRC tumor model after 48 h administration. i) Biodistribution of ZrC@FITC in tumor and the adjacent spleen normal tissues with a metastatic CRC tumor model after 48 h administration. Bar graphs show the mean ± SD. *p < 0.05, **p < 0.01 and ***p < 0.001.

HEK293‐T (human kidney cells), SMMC‐7721 (human liver cancer cells) and A549 (human lung cancer cells) were incubated with ZrC NSs at various concentrations (0, 25, 50, 100, and 200 µg mL^−1^) for 48 h. As shown in Figure 3a, the ZrC NSs showed negligible cytotoxicity in terms of cell viability even at a high concentration of 200 µg mL^−1^. SMMC‐7721 cells were incubated with ZrC NSs for 4 h at 100 µg mL^−1^ and then exposed to an 1064 nm laser at various power densities (0, 0.25, 0.5, 1.0, 1.5, and 2.0 W cm^−2^) for 8 min. Results showed that with increasing laser power density, more cells incubated with ZrC NSs were killed compared with those of the control group incubated without ZrC NSs (Figure 3b). Furthermore, SMMC‐7721 cells were incubated with ZrC NSs at various concentrations (0, 25, 50, 100, and 200 µg mL^−1^) for 4 h and then exposed to the 1064 nm laser (1.0 W cm^−2^, 8 min). The photothermal ablation effect of ZrC NSs is significantly enhanced with elevated ZrC concentrations (Figure 3c). Additionally, SMMC‐7721 cells treated with various concentrations (0, 25, 50, 100, and 200 µg mL^−1^) after ablation were stained and analyzed with acridine orange/propidium iodide staining (live cells, green fluorescence; dead cells, red fluorescence). As the majority of SMMC‐7721 cells were killed gradually by photothermal ablation with increased concentrations of ZrC NSs, the number of dead cells marked by red fluorescence increased correspondingly (Figure 3d). A SN38‐Nif prodrug designed to inhibit tumor cell multiplication and angiogenesis by combining inhibitors of topoisomerase I (SN38) and COX‐2 protein (Nif) was loaded onto ZrC NSs, referred to as ZrC@prodrug. PEGylated ZrC NSs, ZrC@SN38, ZrC@Nif, and ZrC@Iri were prepared as control groups, and their antitumor effects against SMMC‐7721 cells in vitro were studied, as well as those of bare SN38, Nif, Iri and prodrug individually (Figure 3e,f). In SMMC‐7721 tumor‐bearing nude mice, the biodistribution of ZrC NSs (10 mg kg^−1^) in main organs and tumors was investigated, which showed that ≈1.59% of ZrC accumulated in the tumor through the EPR effect at 48 h (Figure 3g). The pharmacokinetics profiles of ZrC@cy7 and ZrC@prodrug‐cy7 NSs at different time were analyzed to measure the NSs concentrations in blood by fluorometry (Figure S8, Supporting Information). Furthermore, results showed that ZrC@FITC had a higher biodistribution (10 mg kg^−1^) in orthotopic tumor tissues than the adjacent normal liver and spleen tissues with a wild‐type MC38 cells‐induced colorectal liver metastasis (CRLM) model^[^ ^33^ ^]^ (Figure 3h,i). These results demonstrated the remarkable biocompatibility, photothermal effect, toxicology in vitro, and biodistribution of the ZrC NSs in promoting cancer ablation.

A detailed investigation of their in vivo toxicology was conducted for further biomedical translation potential. C57BL/6 mice were divided into four groups on the basis of various experimental conditions: 1) control group, 2) mice intravenously injected with PEGylated ZrC NSs (10 mg kg^−1^), 3) mice intravenously injected with ZrC@Iri (10 mg kg^−1^), and 4) mice intravenously injected with ZrC@prodrug (10 mg kg^−1^). The normal haematological parameters, including white blood cells (WBC), red blood cells (RBC), hemoglobin (HGB), mean corpuscular volume (MCV), mean corpuscular hemoglobin (MCH), mean corpuscular hemoglobin concentration (MCHC), haematocrit (HCT) and platelets (PLT), were measured (Figure S9a, Supporting Information). Except for PLT on 7th days in ZrC@prodrug groups, no meaningful changes were observed in the PEGylated ZrC and ZrC@prodrug groups at different time points in comparison to the control group, and the level of PLT on 30th days recovered to the normal level. Results demonstrated that PEGylated ZrC and ZrC@prodrug caused no significant hematological toxicity in mice. Standard blood biochemical indexes were obtained, and various markers, including alanine transaminase (ALT), globulin (GLB), bilirubin (TBIL), total protein (TP), aspartate transaminase (AST), and albumin (ALB), were examined (Figure S9a, Supporting Information). Even though higher expression of TP and ALB was measured in ZrC and ZrC@prodrug groups, it had no significant relationship with cell toxicology, and other indexes in different groups showed no substantial abnormality. Urine creatinine (Cr) and urea are functional indexes for the kidney and there was no significant change between the different groups (Figure S9a, Supporting Information), indicating that PEGylated ZrC and its loaded prodrug induced no significant renal toxicity in mice. Furthermore, major organs (including the heart, liver, spleen, lung and kidney) and bone marrow, were sliced for H&E staining (Figure S9b,c, Supporting Information). No significant acute or chronic pathological toxicity and changes of diastolic and systolic blood pressure, body weight and food intake (Figure S9d, Supporting Information) were found during the treatment period for all groups. These results indicate that PEGylated ZrC and the loaded prodrug have high biocompatibility and can be further evaluated for in vivo cancer treatment.

To evaluate the potential of the photothermal/chemotherapeutic nanoplatform of ZrC@prodrug in cancer PTT in vivo, SMMC‐7721‐induced liver cancer model was established and an approved SN38‐based prodrug (Iri) was used for the positive control. Mice were randomly divided into four groups with established tumors, and aliquots (100 mL) of PBS, PEGylated ZrC, ZrC@Iri, ZrC@prodrug (10 mg kg^−1^) were intravenously injected in mice. At 24 h post‐injection, the entire region of the tumor was irradiated with the 1064 nm laser (1 W cm^−2^) for 8 min. To monitor the photothermal effects in vivo, the changes of the tumor temperature (Δ*T*) were recorded (**Figure** **4**a). Tumor temperature of the mice injected with ZrC, ZrC@Iri, and ZrC@prodrug increased by 20–25 °C, which is significantly higher than that of the control groups (Δ*T* = 5 °C). After the above photothermal treatments with different samples, the same treatments were performed again on the 7th day. As shown in Figure 4b, the ZrC NSs exhibited excellent photothermal efficacy against tumors. The synergistic effect of SN38 (the active form of Iri) in combination with Nif demonstrated a remarkable inhibition of tumor growth without abnormal body weight changes (Figure 4b,c). Highly significant necrosis of tumor cells in the ZrC, ZrC@Iri and ZrC@prodrug groups were checked compared to the control, and ZrC@prodrug and ZrC@Iri induced a higher cell apoptosis rate (TUNEL) with lower cell proliferative activities (Ki67) in tumor compared to the control (Figure 4d), indicating ZrC@prodrug had a similar anti‐tumor effect compared with ZrC@Iri. Lastly, the same experimental procedure was carried out in Hepa1‐6‐induced liver tumor‐bearing mice (C57BL/6), results showed that ZrC@prodrug had a better photothermal/chemotherapeutic treatment effect and dramatically inhibited tumor growth, when it compared with the control, only SN38 (ZrC@SN38) or only Nif‐carried (ZrC@Nif) biophotonic nanoplatforms (Figure 4e).

**Figure 4:**
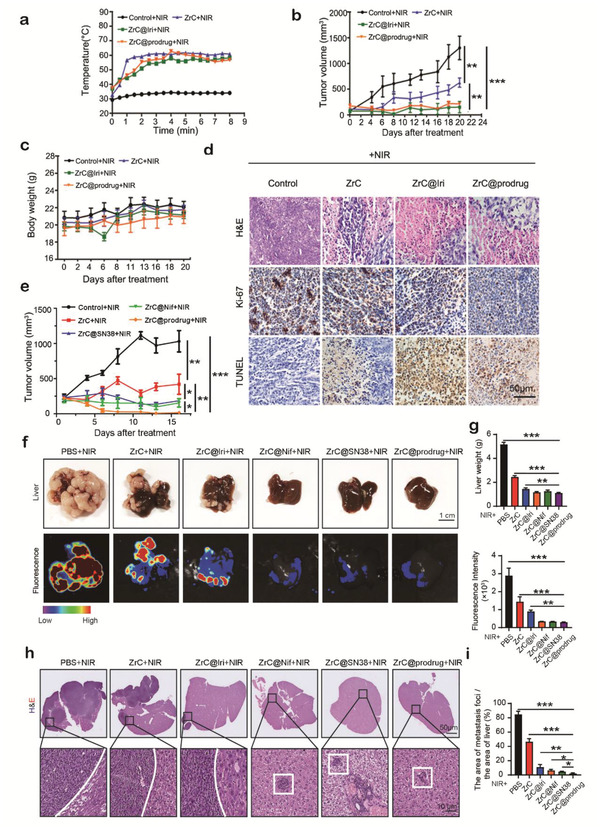
In vivo synergistic inhibition of tumor growth with ZrC@prodrug. a) Temperature curves at the tumor site with liver cancer model after different treatment (control+NIR, PEGylated ZrC+NIR, ZrC@Iri+NIR, and ZrC@prodrug+NIR) at different time intervals (1 W cm^−2^, 8 min). b) Time‐dependent tumor growth and c) body‐weight curves (*n* = 5, mean ± SD) after different treatments in SMMC‐7721‐induced subcutaneous liver cancer models. The NIR treatments were performed twice after 7 days. d) H&E staining, antigen Ki‐67 immunofluorescence, and TUNEL staining for pathological changes in tumor tissues. e) Time‐dependent tumor growth in Hepa1‐6‐induced subcutaneous liver cancer models in C57BL/6 mice with different treatments. f) 2 weeks later after different treatments (control+NIR, PEGylated ZrC+NIR, ZrC@Iri+NIR, ZrC@Nif+NIR, ZrC@SN38+NIR, and ZrC@prodrug+NIR), images of tumors in liver. g) Quantification of liver weight and fluorescence intensity in liver after various treatments. h) H&E staining in liver after various treatments. i) Percentage (%) of the area of metastasis loci in liver tissues after various treatments.

Besides the primary tumor, we also investigated the therapeutic effect of ZrC@prodrug‐mediated photothermal/chemotherapeutic tumor eradication for metastatic tumors with the presence of host immunity. Colorectal liver metastasis (CRLM) model was established in C57BL/6 mice using MC38‐GFP colorectal cancer cells. When compared with control, ZrC and ZrC@Iri treated groups, ZrC@prodrug‐mediated therapy had a better anti‐tumor effectiveness in the CRLM model measured by liver weight and fluorescence intensity (Figure 4f,g). Compared to the other groups (included ZrC@SN38 and ZrC@Nif +NIR treated groups), a smaller number of metastatic nodules (foci) was detected in livers after ZrC@prodrug‐mediated combined therapy in CRLM model (Figure 4h,i).

Conventional chemotherapy and PTT pose several challenges, including chemotherapy‐ and hyperthermia‐induced inflammation, angiogenesis, and upregulation of growth factors and cytokines for tumor recurrence and metastasis.^[^ ^21^, ^27^ ^]^ In addition to the known impact of PTT on malignant cells, we further explored the effect of ZrC@prodrug‐mediated PTT on changing tumor microenvironment (TME). For this purpose, we analyzed the remaining tumors tissues after various treatments in the CRLM model using wild type‐MC38 cells. As shown in **Figure** **5**a, signals of dramatical reduction of Ki67^+^ colorectal cancer cell proliferation, increase of C‐caspase3^+^ tumor cellular apoptosis and CA9+ hypoxia, and regression of CD31+ vasculatures, were detected. Furthermore, the CD4+ and CD8+ cell population and tumor‐associated macrophages (F4/80+) in tumor tissues were decreased after treatment of ZrC@prodrug, implying that the intratumor immune response was also altered in TME. The results indicate that prodrug‐based PTT induces malignant cell death and substantially alters TME.

**Figure 5:**
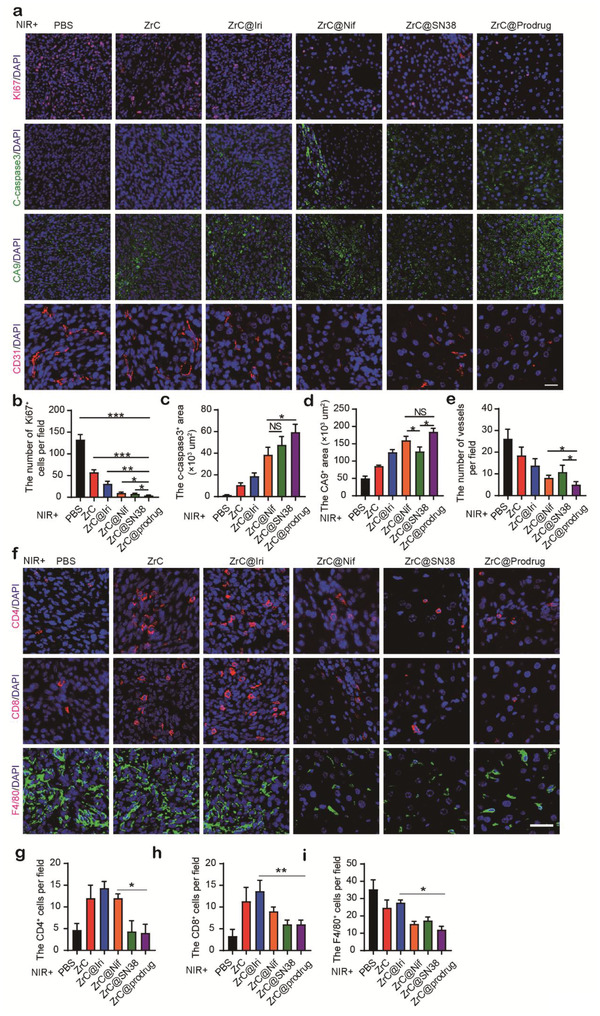
Synergistic inhibition of tumor growth with dual targeting malignant cells and tumor stroma. Tumors tissues in colorectal liver metastasis (CRLM) model, were isolated 14 days after various treatments, and a) representative immunofluorescence staining analysis of Ki67, C‐caspase3, CA9 and CD31 in tissues were obtained for the changes in the tumor environment (TME). Statistical analysis of a functional parameter type, such as b) cancer cell proliferation by Ki67, c) cellular apoptosis by C‐caspase3, and d) hypoxia signal by CA9+, and e) regressive vasculature by CD31. f) Representative immunofluorescence staining analysis of CD4, CD8, and F4/80 in CRLM tumor tissues. g) Statistical analysis of CD4, CD8, and F4/80‐positive cells in each field. *n* > 5 fields from four tumors. Bar graphs show the mean ± SD. *p < 0.05, **p < 0.01, and ***p < 0.001. ns: no significance. Scale bar, 50 µm.

Irinotecan (Iri) is an approved clinical drug, and Iri and SN38‐Nif both are a kind of SN38‐based prodrug. To further distinguish advantages between Iri and SN38‐Nif, we employed a visceral colorectal cancer (CRC) tumor model in our study using MC38 cells. Fluorescence‐activated cell sorting (FACS) analysis with a panel of specific markers was used to define various populations of inflammatory and immune cells. ZrC‐mediated PTT showed a significant increases in the total CD45^+^ population (Figure S10a, Supporting Information); the CD45^+^/B220^+^ population (B cells); the CD45^+^/CD3^+^/CD4^+^ population (CD4^+^ T cells); the CD45^+^/CD3^+^, the CD8^+^ population (CD8^+^ T cells); the CD45^+^/MHCII^+^/CD11b^+^ population (myeloid cells); the CD45^+^/MHCII^+^/CD11b^+^/CD11c^+^ population (myeloid cells); the CD45^+^/NK1.1^+^ population (natural killer cells); the CD45^+^/Ly6C^+^ population (granulocytes); the CD45^+^/Ly6G^+^ population (granulocytes); and the CD45^+^/CD11b^+^/F4/80^+^/CD206^+^ population (M2‐like macrophages) (Figure S10a–d, Supporting Information). These results demonstrated that PTT markedly increased infiltration of inflammatory cells in tumors, which might contribute to protumorigenic and metastatic phenotypes in the tumor microenvironment.^[^ ^25^, ^27^ ^]^ In comparison to the ZrC and ZrC@Iri groups, ZrC@prodrug group exhibited significantly reduced infiltration of inflammatory cells (Figure S10a,d, Supporting Information). The underlying mechanism was most likely due to blocking the COX2‐induced inflammatory effect and vascularization‐permitted inflammatory cell infiltration

Moreover, in the CRLM model, haemoglobin oxygen saturation (sO2) status was detected by photoacoustic imaging.^[^ ^34^ ^]^ In comparison with ZrC and ZrC@Iri groups, the ZrC@prodrug group showed a further decrease of sO2‐rich area in tumors, validating a potent antiangiogenic effect of the prodrug‐converted products (**Figure** **6**a). Notably, similar reduction of sO2‐rich area was also detected in surrounding liver tissues (Figure 6b). In addition to SN38‐induced cancer death by inhibiting DNA replication, we hypothesized that simultaneous targeting of COX‐2 and VEGF pathways might improve the antiangiogenic activity after SN38‐Nif prodrug conversion. To test this hypothesis, SMMC‐7721 and LLC cells were cultured with PEGylated ZrC, ZrC@Iri, ZrC@prodrug for 48 h in vitro at various concentrations (10 and 20 µg mL^−1^). Protein levels of COX‐2, PGE_2_, and VEGF protein were analyzed. COX‐2, PGE_2_, and VEGF were markedly downregulated in the ZrC@prodrug group in vitro (Figure 6c). Interestingly, a low dose of PEGylated ZrC and ZrC@Iri at a concentration of 10 µg mL^−1^ slightly increased expression of VEGF compared to that at a concentration of 20 µg mL^−1^ in LLC cells. Furthermore, after treatment with ZrC@prodrug‐induced photothermal and chemotherapeutic combination therapy, expression levels of CD31, COX‐2, PGE_2_, and VEGF in the SMMC‐7721 liver cancer were evaluated. Microvessel density was examined by immunohistochemical staining with CD31, which was markedly reduced in the ZrC@prodrug‐treated group than in the PEGylated ZrC and ZrC@Iri control groups. Similarly, decreases of COX‐2, PGE_2_, and VEGF were also seen in the ZrC@prodrug‐treated group (Figure 6d). Collectively, these results provide mechanistic insights on the newly designed combination therapy, suggesting that ZrC@prodrug inhibits tumor growth by targeting tumor cells, inflammation, and angiogenesis.

**Figure 6:**
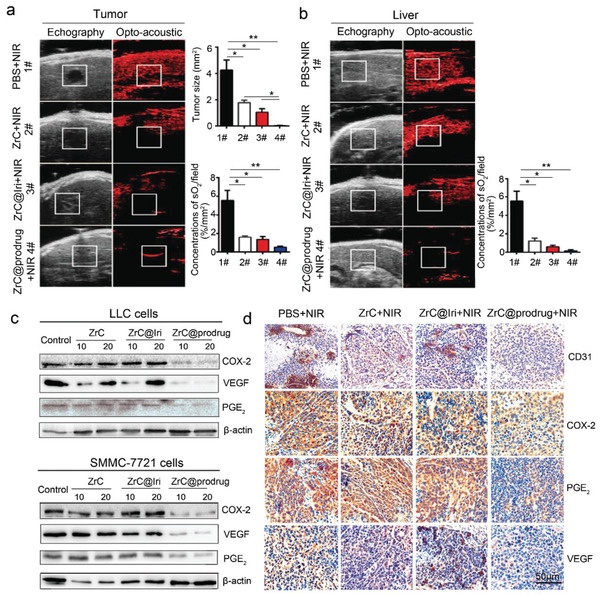
Tumor anti‐angiogenesis of ZrC@prodrug through the COX‐2/PGE_2_/VEGF pathway. a) Photoacoustic and ultrasound images, and quantifications of hepatic metastases in mice after various treatments. b) Photoacoustic and ultrasound images and quantifications of tumor surrounding liver tissue in mice after various treatments. c) Western blot analysis of COX‐2, PGE_2_, and VEGF in treated cells. In vitro tumor anti‐angiogenesis in the control, ZrC (PEGylated ZrC), ZrC@Iri, ZrC@prodrug (10 and 20 µg mL^−1^) when incubated with LLC cells and SMMC‐7721 cells individually for 48 h. d) Immunohistochemical staining of CD31, COX‐2, PGE_2_, and VEGF in treated tumor tissues with × 20 magnification. Liver (SMMC‐7721 cells) tumors were collected from mice on the 20th day after NIR treatment with control, PEGylated ZrC, ZrC@Iri, ZrC@prodrug (10 mg kg^−1^) with tail vein injection. All images share the same scale bar (50 µm). NIR: near‐infrared radiation. Bar graphs show the mean ± SD. *p < 0.05, **p < 0.01, and ***p < 0.001.

A promising type of ultrathin ZrC NSs with efficient photothermal performance in NIR‐II biowindow has been demonstrated for the first time. We synthesized and loaded an anticancer prodrug (SN38‐Nif) onto ZrC NSs (ZrC@prodrug). The prodrug is composed of SN38 and Nif, which can be released under the photothermal effect of ZrC NSs and selectively activated by commonly tumor‐overexpressed esterase.^[^ ^26^ ^]^ Consequently, the prodrug‐loaded ZrC biophotonic nanoplatform delivers prodrugs to tumors by generating EPR effect after esterase cleavage and NIR light excitation. This design produces synergistic anti‐tumor effects with chemotherapy. Our approach has several advantages over existing photothermal nanomaterials, including 1) Conversion of two prodrugs into two active antitumor compounds by targeting tumor cells, inflammation and angiogenesis; 2) Tumor specific targeting by carboxylesterase. Since the carboxyl esterase is particularly abundant in malignant cells but not normal cells, tumor cell‐ specific targeting is achieved cleaving the prodrug in tumor cells, but not in normal cells; 3) Zirconium carbide nanosheets is a completely new 2D photothermic nanomaterial, which exhibits more effective light absorption and high efficiency of light‐to‐heat conversion; 4) In addition to targeting tumor cells, the prodrug cleavage‐released Nif also targets tumor inflammation and angiogenesis, two important processes for supporting tumor growth and metastasis; 5) Multiple anticancer mechanisms. Our approach provides at least three anticancer mechanisms: i) Zirconium carbide particle‐generated heat; ii) a DNA topoisomerase inhibitor SN38; and iii) a COX2 inhibitor; Targeting both cancer cells and stromal cells in the tumor microenvironment; and 6) The existing light‐based thermotherapy often augments an inflammatory response. Using the prodrug approach, tumor inflammation is virtually ameliorated.

In materials science, the synthesized ZrC shows much higher PTCE than Au NRs, BPQDs, and Ti_3_C_2_ NSs. In addition, compared with semiconducting BPQDs, the metallic ZrC with plasmonic effect contributes to higher PTCE.^[^ ^16^ ^]^ The spin‐orbit coupling of a photothermal agent can be enhanced when there is a heavy atom in its constituent, which is called external heavy atom effect. Through employing this effect, the problem of spin prohibition of the photothermal agent can be solved. Therefore, the photothermal conversion ability of photothermal agent with external heavy atom effect can achieve enhanced performance. Zr possesses a larger number of atoms than titanium, and thus ZrC shows more efficient photothermal conversion than Ti_3_C_2_ owing to the external heavy atom effect.^[^ ^17^, ^35^ ^]^ Furthermore, the specific electronic structure of early transition metal leads to highly efficient photothermal conversion than noble metals, such as the Au.^[^ ^30^ ^]^ Our results show that ≈1.59% ID g^−1^ of ZrC accumulated in the tumor through the EPR effect, and the high signal found in the liver is similar to most of the reported inorganic materials, such as Nb2C‐PVP NSs (2.24%)^[^ ^36^ ^]^ and Ti3C2 (1.74%).^[^ ^16^, ^32^ ^]^ Interestingly, the temperature of the tumor site under the laser irradiation is sufficiently high to kill most cancer cells. ZrC NSs had a higher biodistribution in orthotopic tumor tissues than the adjacent liver and spleen normal tissues with a metastatic CRC tumor model. In addition, both ZrC NSs and PEGylated ZrC NSs show excellent dispersion and stability in physiological medium, due to the hydrophilic and negatively charged surface. Though prodrug and PEG polymer were loaded on the surface of ZrC NSs by the van der Waals and H‐bond interactions, the PEG polymer bond to ZrC more firmly compared with prodrug, due to the large molecular weight.

Most current available cancer therapeutics are designed by targeting a particular signaling pathway either directly on tumor cells or stromal cells. Although conventional therapeutics such as chemotherapy are effective, these therapeutic approaches often encounter recurrence of tumors and even metastasis. Moreover, malignant cells often develop drug resistance toward these therapeutic agents through multiple mechanisms. Tumor inhibition by conventional chemotherapy and radiation therapy is usually associated with the tumor microenvironment reprogramming and treatment failure. Consequently, enhanced tumor inflammation and angiogenesis due to hypoxia and mechanical stress would facilitate tumor regrowth and metastasis. Currently, these clinically relevant issues remain unresolved. Here in this study, we aim to target malignant cells, tumor inflammation, and angiogenesis thorough a single approach. The combination of photothermal heat, chemotherapeutics and prodrug‐generated products almost completely eradicated the tumors in preclinical models. Importantly, no tumor recurrence was observed with our powerful anti‐tumor approach. This multiple combination approach by combining photo‐physics, chemistry, and biologics for combating cancers is extremely effective.

Another interesting aspect of our approach is tumor specific targeting. While most cancer specific targeting approaches emphasize defining tumor cell specific markers, we have taken the advantage of the high expression of carboxylesterase in malignant cells, but not in healthy non‐tumoral cells. Moreover, our approach is not to block this enzymatic activity, rather to use this enzyme for generating active anticancer products. Carboxylesterase is often elevated to high levels in most types of malignant cells and thus our approach can be generalized to treat many different cancers. The prodrug‐converted final products produce a spill‐out anticancer effect by targeting the tumor microenvironmental components, including angiogenesis and inflammation. Angiogenesis and inflammation are essential for cancer metastasis and dormant tumor regrowth. Blocking these two processes are tremendously important for increasing therapeutic efficacy.

The major hurdles that hamper photothermal nanomaterials for clinical use are relatively low therapeutic efficacy, adverse effects, and biocompatibility. In this study, we provide evidence to show that the ZrC@prodrug is a highly potent antitumor approach, nontoxic, and biocompatible. This approach circumvents the obstacles toward clinical development. Although it is not yet tested, it is reasonably speculated that our ZrC@prodrug approach with other anticancer therapeutics, especially the targeted therapy approaches would produce even greater anticancer effects. It is likely that combination of our approach with other therapeutic modalities would eradicate tumors. Particularly, our approach is also effective for treating metastatic cancers. Taken together, the ZrC@prodrug approach has brought a new hope for effectively treating cancers and warrants clinical trials.

## Experimental Section

##### Materials

The bulk ZrC powder was purchased from a commercial supplier (Smart‐Elements, Austria) and stored in a dark argon glovebox. Isopropanol (IPA) and ethanol (99.5%, anhydrous) were purchased from Aladdin Reagents (Shanghai, China). PEG‐NH_2_ was purchased from Shanghai Yare Biotech, Inc. (China). Esterase was purchased from Sigma Aldrich. The AO/PI Assay Kit was obtained from Logos Biosystems (Korea). PBS (pH 7.4), FBS, RPMI‐1640 medium, trypsin‐EDTA, and penicillin/streptomycin were purchased from Gibco Life Technologies. All other chemicals used in this study were of analytical reagent grade and used without further purification. Ultrapure water (18.25 MΩ cm^−1^, 25 °C) was used to prepare all of the solutions.

##### Synthesis of ZrC NSs

The ultrathin ZrC NSs were prepared from non‐layered bulk ZrC using LPE and were obtained by two steps of probe sonication and bath sonication. Specifically, 100 mg of ZrC powder was mixed with 100 mL of IPA. The mixture underwent probe sonication for 8 h at 200 W. To avoid thermal oxidation during the sonication process, the probe sonication was set to an on/off cycle of 2/2 s, and the ZrC NS suspension was kept in ice water. Subsequently, the ZrC NS suspension was subjected to bath sonication at 360 W for a further ≈10 h. The water bath temperature was maintained at 10 °C. After these two sonication processes, the prepared dispersions were centrifuged at 1000 g for ≈30 min to remove the large ZrC particles. The supernatant containing the ZrC NSs was decanted gently and then further centrifuged for 30 min at 8000 g. The ZrC NSs were dried in a vacuum drying oven, packaged in tinfoil and stored at 4 °C.

##### Synthesis of Prodrug

Niflumic acid (110 mg, 0.38 mmol) was added to a stirred solution of EDC·HCl (100 mg, 0.51 mmol) and DMAP (130 mg, 1.02 mmol) in DCM (10 mL) at 0 °C. After 1 h, SN38 was added and stirred for 12 h at room temperature. The reaction was monitored by TLC. After completion of reaction, the reaction mixture washed with 1 n HCl, water, dried over anhydrous Na_2_SO_4_ and concentrated under reduced pressure. The crude product was purified by column chromatography on silica gel using 2–4% methanol in DCM as eluent to obtain SN38‐Nif as pale yellow green solid (110 mg; 65.7%). The analytical data (1H NMR, 13C NMR) for SN38‐Nif was fully consistent with the proposed structure (Figures S11 and S12, Supporting Information).

##### Synthesis of ZrC@prodrug

i) Synthesis of ZrC‐PEG‐NH_2_ nanoparticles (PEG‐ZrC): PEG‐ZrC nanoparticles were prepared by electrostatic adsorption. Briefly, PEG‐NH_2_ and ZrC NSs were mixed in water at a ratio of 2:1 and stirred at room temperature for 30 min. ii) Synthesis of ZrC@prodrug: ZrC‐PEG‐NH_2_ was loaded with prodrug by electrostatic adsorption. ZrC‐PEG‐NH_2_ was dispersed in water at 1 mg mL^−1^, and the prodrug was dissolved in DMSO at 10 mg mL^−1^. The ZrC‐PEG‐NH_2_ dispersion was mixed with the prodrug solution at a mass ratio of 2:1. The resulting mixture was stirred at room temperature for 30 min and washed with PBS 3 times.

##### Morphology and Characterization

TEM and HRTEM were employed to characterize the morphology and elemental composition of ZrC NSs. SAED and energy‐dispersive X‐ray spectrometry (EDS) were performed at 300 kV by using a FEI Tecnai G2 F30 field‐emission TEM instrument. AFM was employed to characterize the morphology and height of ZrC NSs by using a BRUKER Dimension Fastscan, with the samples dispersed on Si/SiO_2_ substrates by a drop‐casting method and the images recorded at 512 pixels per line. XPS was performed to analyze the surface chemicals of ZrC NSs on an ULVAC PHI 5000 Versa Probe II using an Al K*α* (*λ* = 0.83 nm, *hυ* = 1486.7 eV) X‐ray source operated at 23.5 W, and the data were analyzed by MultiPak Version 9.0 software. The Raman spectrum was acquired by the InVia Reflex confocal Raman microscope equipped with a 532 nm argon ion laser as the excitation source. Fourier transform infrared (FTIR) spectra were used to confirm the ZrC NSs and the ZrC@prodrug. UV–vis spectroscopy was performed using a HITACHI UH4150 spectrophotometer to measure the optical absorbance of ZrC NSs in the range of 400–1100 nm.

##### In Vitro Cytotoxicity Assay of ZrC NSs

Cells from the human kidney HEK293‐T cell line (denoted as HEK293‐T cells; Cell Bank of Shanghai Institutes for Biological Sciences, Chinese Academy of Sciences), the human lung cancer A549 cell line (denoted as A549 cells; Cell Bank of Shanghai Institutes for Biological Sciences, Chinese Academy of Sciences) and the human liver cancer SMMC‐7721 cell line (denoted as SMMC‐7721 cells; Cell Bank of Shanghai Institutes for Biological Sciences, Chinese Academy of Sciences) were used for the in vitro cytotoxicity assay of ZrC NSs. Cells were cultured with Dulbecco's modified Eagle's medium (DMEM, Gibco, Invitrogen) under 5% CO_2_ and supplemented with 10% fetal bovine serum (FBS, Gibco, Invitrogen) and 1% penicillin/streptomycin in a humidified incubator at 37 °C. After harvesting with 0.25% trypsin‐EDTA solution (cat. no. C0201, Beyotime, China), cells were seeded in 96‐well culture plates (Corning) at a density of 1 × 10^5^ cells per well (*n* = 4) for 24 h, and the culture medium was then replaced with fresh culture medium containing ZrC NSs at different concentrations (0, 25, 50, 100, and 200 µg mL^−1^). After 24 h of incubation, the in vitro cytotoxicity of ZrC NSs was determined by the CCK‐8 viability assay (Cell Counting Kit, Dojindo Laboratories, Kumamoto, Japan).

##### In Vitro Photothermal/Chemotherapeutic Tumor Eradication of ZrC NSs and ZrC@prodrug

i) To examine the photothermal effect of ZrC NSs at various power densities in vitro, SMMC‐7721 cells were seeded in a 96‐well plate at a density of 1 × 10^5^ cells per well (*n* = 4/group) in DMEM at 37 °C with 5% CO_2_ for 8 h before treatment. The culture medium was replaced with DMEM and ZrC NSs (100 µg mL^−1^, 100 µL per well). After 4 h of incubation, the medium and unbound NSs were removed, and the cells were washed three times with PBS (Hyclone, Thermo Scientific Biotech Co., Ltd., USA), after which fresh medium was added into the wells. SMMC‐7721 cells were then irradiated for 8 min under a 1064 nm laser at various power densities (0, 0.25, 0.5, 1.0, 1.5, and 2.0 W cm^−2^). Finally, a CCK‐8 assay was performed to evaluate the viability of cells after 12 h. ii) To examine the photothermal effect of ZrC NSs at different concentrations in vitro, SMMC‐7721 cells were seeded in a 96‐well plate at a density of 1 × 10^5^ cells per well overnight, and complete medium containing different concentrations of ZrC NSs (0, 25, 50, 100, and 200 µg mL^−1^, 100 µL) was added to the wells. Cells were incubated for 4 h, and then each well was irradiated using a laser (1064 nm, 8 min, 1.0 W cm^−2^). Finally, the viability of cells (*n* = 4/group) after 12 h was evaluated using a CCK‐8 assay, and the absorbance at 450 nm was determined with a microplate spectrophotometer (Varioskan Flash 4.00.53, Finland). The cell viability was normalized using the following formula: cell viability (%) = (mean of Abs. value of treatment group/mean of Abs. value of control) × 100%. The control group was cells without any treatment. iii) SMMC‐7721 cells that were co‐cultured with 100 µg mL^−1^ ZrC NSs and then treated with the laser (1064 nm, 1.0 W cm^−2^, 8 min) were gently washed three times with PBS after 12 h and then incubated with Calcein‐AM and PI solution (Dojindo Laboratories, Kumamoto, Japan) for 15 min according to the manufacturer's instructions. Living cells (green fluorescence) and dead cells (red fluorescence) were distinguished using an Olympus IX71 inverted fluorescence microscope. iv) To examine the chemotherapeutic anti‐tumor effect of ZrC@prodrug at different concentrations in vitro, SMMC‐7721 cells were seeded in a 96‐well plate at a density of 1 × 10^5^ cells per well overnight, and complete medium containing different concentrations of prodrug, ZrC NSs, ZrC@Iri and ZrC@prodrug (0, 25, 50, and 100 µg mL^−1^, 100 µL) was added to the wells. The viability of cells (*n* = 4/group) after 48 h was evaluated using a CCK‐8 assay.

##### In Vivo Toxicity, Pharmacokinetic and Biodistribution Analysis

Mice were purchased from the Shanghai SLAC Laboratory Animal Co., Ltd. (license no. SCXK (HU) 2007‐0003; Shanghai, China) and were housed in specific pathogen‐free (SPF) conditions with a 12‐h light cycle and with food and water ad libitum. i) For the in vivo toxicity assay of ZrC NSs, twenty healthy female C57BL/6 mice (5–6 weeks old, 16–20 g) were randomly divided into four groups (*n* = 5/group). The mice were intravenously injected with ZrC at various concentrations in PBS (0, 1, 5, and 10 mg kg^−1^). After 28 days of feeding, the major organs (heart, liver, spleen, lung, and kidney) and bone marrow in different groups were harvested for H&E staining. ii) For the in vivo toxicity assay of ZrC@prodrug, 48 healthy female C57BL/6 mice (5–6 weeks old, 16–20 g) were divided randomly into four groups (*n* = 12/group): 1) the control group (saline, 100 µL), 2) mice intravenously injected with PEG‐ZrC (10 mg kg^−1^, 100 µL), 3) mice intravenously injected with ZrC@Iri (10 mg kg^−1^, 100 µL), and 4) mice intravenously injected with ZrC@prodrug (10 mg kg^−1^, 100 µL). The histological, hematological, urine and blood biochemical indexes were assessed at various time intervals (1st, 7th, and 30th days) after intravenous administration. Approximately 0.8 mL of blood (*n* = 4/group), 0.4 mL of serum (*n* = 4/group) and 0.2 mL of urine (*n* = 4/group) were collected from each mouse and diluted with saline solution (2 times dilution) for assay at the Shenzhen Sun Yat‐sen Cardiovascular Hospital. Meanwhile, mice were tested for conscious systolic blood pressure (SBP) and diastolic blood pressure (DBP) using the Kent Scientific Coda BP System (Torrington, CT).

In the in vivo pharmacokinetic analysis, C57BL/6 mice were intravenously injected with ZrC@cy7 or ZrC@prodrug‐cy7 (100 µL, 1 mg mL^−1^). 100 µL orbital blood was obtained at certain time and dissolved in 1 mL of the lysis buffer. A multifunctional fluorometer (Thermo Scientific Varioskan LUX) was used to measure the concentration of cy7 in the blood. The control (blank blood samples) and a standard calibration curve were made.

In the in vivo biodistribution analysis of ZrC, subcutaneous liver tumors were established with SMMC‐7721 cells in Balb/c nude mice. Biodistribution of Zr (% ID of Zr per gram of tissues) in main tissues and tumors after the intravenous administration of ZrC dispersed in PBS at 48 h were analyzed using the Inductive Coupled Plasma Emission Spectrometer (ICP) technique (Perkin Elmer). For fluorescence imaging experiments, In the Balb/c nude mice with the MC38 cells‐induced CRC liver metastasis model were intravenously injected with the Cy5.5‐labelled ZrC/ PLGA NSs (100 mL of 1 mg mL^−1^ for each mouse) and examined by a fluorescence (Xenogen IVIS‐Spectrum) imaging system as a function of time for up to 48 h.

##### In Vivo Tumor Photothermal/Chemotherapeutic Therapy

i) To establish a subcutaneous human liver cancer model, twenty healthy female BALB/c nu/nu mice (5–6 weeks old, 16–20 g) were used. A total of 5 × 10^6^ human liver cancer SMMC‐7721 cells (denoted as SMMC‐7721 cells; Cell Bank of Shanghai Institutes for Biological Sciences, Chinese Academy of Sciences) were injected subcutaneously into the right flank of the mouse. When the tumor volume reached ≈200 mm^3^, all mice were randomly divided into four groups (*n* = 5/group): 1) the control group (saline, 100 µL), 2) mice intravenously injected with PEGylated ZrC (10 mg kg^−1^, 100 µL), 3) mice intravenously injected with ZrC@Iri (10 mg kg^−1^, 100 µL), and 4) mice intravenously injected with ZrC@prodrug (10 mg kg^−1^, 100 µL). Twenty‐four hours later, the mice were anaesthetized with pentobarbital (50 mg kg^−1^) through intraperitoneal injection, and in vivo photothermal therapy was performed with NIR laser irradiation (1 W cm^−2^, 8 min). After the above photothermal treatments with different samples, the same treatments were performed again on 7th day. The measurements of the tumor volumes were then taken with a digital calliper every 2 days for 2 weeks. ii) To establish a subcutaneous mouse liver cancer model, healthy female C57BL/6 mice (5–6 weeks old, 16–20 g) were used. A total of 5 × 10^5^ mouse liver cancer Hepa1‐6 cells (denoted as Hepa1‐6 cells; Cell Bank of Shanghai Institutes for Biological Sciences, Chinese Academy of Sciences) were injected subcutaneously into the right flank of the mouse. When the tumor volume reached ≈200 mm^3^, all mice were randomly divided into five groups (*n* = 5/group): 1) the control group (saline, 100 µL), 2) mice intravenously injected with PEGylated ZrC (10 mg kg^−1^, 100 µL), 3) mice intravenously injected with ZrC@Nif (10 mg kg^−1^, 100 µL), 4) mice intravenously injected with ZrC@SN38 (10 mg kg^−1^, 100 µL), and 5) mice intravenously injected with ZrC@prodrug (10 mg kg^−1^, 100 µL). Twenty‐four hours later, in vivo photothermal therapy was performed with NIR laser irradiation (1 W cm^−2^, 8 min). The same treatments were performed again on 7th day. iii) To establish a visceral colorectal cancer (CRC) tumor model, a left subcostal surgical incision was created and 1 × 10^6^ MC38 cells in 25 µL of PBS were injected into the exposed hemi‐spleen of each mouse. In vivo photothermal therapy were performed as previously described. Two weeks after tumor implantation, mice were sacrificed and liver were dissected. Volumes of surface visible metastatic nodules were calculated. Tumor GFP signal validation were performed ex vivo using a fluorescent imaging system (VISQUE InVivo Elite, Vieworks, Korea). iv) To establish a colorectal liver metastasis (CRLM) model,^[^ ^33^ ^]^ a spleen injection of 1 × 10^6^ MC38 cells in 25 µL of PBS were performed in each mouse. In vivo photothermal therapy were performed as previously described. Two weeks after tumor implantation, mice were sacrificed and livers were dissected. Volumes of surface visible metastatic nodules were calculated. Metastatic tumor GFP signal validation were performed ex vivo using a fluorescent imaging system (VISQUE InVivo Elite). The tumor volume was measured according to the following formula: tumor volume = (tumor length) × (tumor width)^2^/2. The tumors were dissected and fixed with 4% neutral paraformaldehyde. Thereafter, the tumor issues were sectioned into slices for H&E staining, immunohistochemical staining and the TUNEL assay. Mice whose tumors were larger than 2.0 cm in length were euthanized according to the standard animal welfare guidelines.

##### FACS Analysis

Tumor tissues were dissected and homogenized. Tissue suspension was treated for 3 min with 5 mL of a red blood cell lysis buffer (cat. no. 00‐4333‐57, eBioscience, Inc., Waltham, MA, USA). After PBS washing, cell suspension was fixed for 30 min with 4% PFA. Single‐cell suspensions were incubated with 0.5 mg mL anti‐mouse Fc*γ*III/II receptor antibody (cat. no. 553 141, BD Bioscience, San Jose, CA, USA) for 10 min and were incubated with various conjugated antibodies for 30 min on ice. These antibodies include: a PerCP‐eFluor 710 anti‐mouse MHC Class II antibody (cat. no. 46‐5321‐82, eBioscience); a PE anti‐mouse CD11c antibody (cat. no. 12‐0114‐82, eBioscience); a PE‐Cyanine7 anti‐mouse CD11b antibody (cat. no. 25‐0112‐82, eBioscience); an APC anti‐mouse Ly‐6C antibody (cat. no. 17‐5932‐82, eBioscience); an eFluor 450 anti‐mouse Ly‐6G antibody (cat. no. 48‐9668‐82, eBioscience); an eFluor 506 anti‐mouse CD45 antibody (cat. no. 69‐0451‐82, eBioscience); a PerCP‐Cyanine5.5 anti‐mouse CD8a antibody (cat. no. 45‐0081‐82, eBioscience); a PE anti‐mouse B220 antibody (cat. no. 12‐0452‐82, eBioscience); a PerCP‐Cyanine7 anti‐mouse CD4 antibody (cat. no. 25‐0042‐82, eBioscience); an APC anti‐mouse NK1.1 antibody (cat. no.17‐5941‐82, eBioscience); an eFluor 450 anti‐mouse CD3e antibody (cat. no. 48‐0031‐82, eBioscience); a PE anti‐mouse CD206 antibody (cat. no. 141 706, biolegend); an APC anti‐mouse F4/80 antibody (cat. no. 17‐4801‐82, eBioscience); an eFluor 450 anti‐mouse CD86 antibody (cat. no. 48‐0862‐82, eBioscience); and an eFluor 780 Viability Dye (cat. no. 65‐0865‐14, eBioscience); The stained cells were applied onto FACScan (BD) and analyzed by FlowJo version 10 (Tree Star, Inc., Ashland, OR, USA).

##### In Vivo Photoacoustic Imaging

Ultrasound and photoacoustic imaging were performed using a Vevo LAZR (FUJIFILM VisualSonics, Inc., Canada) imaging system.^[^ ^34^ ^]^ Animals with different ZrC‐mediated treatment were anaesthetized with isoflurane and were setup on a platform that monitored the respiration rate and the heart rate. A 30 MHz, linear array ultrasound transducer with integrated fiber optic light delivery (LZ‐400 and MX‐400, FUJIFILM VisualSonics, Inc.) was positioned longitudinally overtop the liver. Ultrasound imaging was used to make sure that the liver was in the field‐of‐view. The integrated fiber bundle delivered 15 to 20 mJ cm^−2^ of light to the liver of the mouse. The images were acquired by detecting the spectra of the oxygenated hemoglobin at 682 nm.

##### Haematoxylin and Eosin (H&E) Staining, Immunohistochemical Staining and TUNEL Assay

Tumor tissues from mice subjected to different treatments were fixed in 10% neutral buffered formalin, embedded in paraffin, sectioned at 8 mm, stained with H&E, and examined by digital microscopy. For the immunohistochemical staining assay, the paraffin‐embedded sections (tumor tissues) were incubated with anti‐Ki‐67 antibody (cat. no. 27309‐1‐AP; 1:300; ProteinTech, Inc., Rosemont, IL, USA), anti‐caspase‐3 antibody (cat. no. A11319; 1:200; ABclonal, Wuhan, China), anti‐CA9 antibody (cat. no. A1658; 1:200; ABclonal), anti‐CD31 antibody (cat. no. 11265‐1‐AP; 1:1000; ProteinTech), anti‐COX‐2 antibody (cat. no. 12375‐1‐AP; 1:100; ProteinTech), anti‐VEGF antibody (cat. no. 19003‐1‐AP; 1:300; ProteinTech), or anti‐PGE_2_ antibody (cat. no. ab2318; 1:300; Abcam) overnight at 4 °C, followed by incubation with 100 µL of 1 × secondary antibody solution for 1 h (cat. no. KIHC‐5, 1:1, ProteinTech). The signals were detected by staining the sections with 3, 3’‐diaminobenzidine and haematoxylin (ProteinTech).

Cell apoptosis in tumor tissues was detected with the DeadEnd Colorimetric TUNEL System (cat. no. G7130, Promega, Inc., Madison, USA). Briefly, the tumor slides were incubated with terminal deoxynucleotide transferase (TdT) and a biotinylated nucleotide mixture at 37 °C for 30 min. Subsequently, the endogenous peroxidases were blocked by immersing the slides in 0.3% hydrogen peroxide in PBS for 3–5 min at room temperature. The slides were incubated with streptavidin‐HRP and visualized with DAB. Negative controls were set up by substituting distilled water for TdT in the working solution. The results are presented as the ratio of the TUNEL‐positive cells to the total number of cells.

##### Protein Sample Preparation and Western Blotting

A total of 5 × 10^5^ LLC and SMMC‐7721 cells were seeded into 6‐well plates (*n* = 3/group) in DMEM at 37 °C with 5% CO_2_ for 8 h before treatment. The culture medium was then replaced with DMEM (control) or with DMEM containing PEG‐ZrC, ZrC@Iri or ZrC@prodrug nanomaterials individually at concentrations of 10 or 20 µg mL^−1^ (100 µL per well). After 48 h of incubation, the medium and unbound NSs were removed. Total protein samples were extracted from the cells using RIPA lysis buffer (cat. no. P0013B, Byotime, Inc., Shanghai, China), and the concentration was detected by a BCA protein quantitation kit (cat. no. P0010, Byotime). Western blotting was performed. Briefly, cell lysates containing 10 µg of total protein and 1 × loading buffer were separated by standard SDS‐PAGE, followed by transfer to polyvinylidene difluoride membranes (Millipore, Billerica, MA, USA). The membranes were incubated with primary antibodies against VEGF (cat. no. 19003‐1‐AP; 1:1000, ProteinTech), COX‐2(cat. no. 12375‐1‐AP; 1:1000, ProteinTech) and PGE_2_ (cat. no. ab2318; 1:1000, Abcam, Inc., Cambridge, UK) at 4 °C overnight. Next, the membranes were incubated with an appropriate horseradish peroxidase (HRP)‐conjugated anti‐rabbit IgG (cat. no. 7074; 1:2000, Cell Signalling Technology, Inc., Danvers, MA, USA) or HRP‐conjugated anti‐mouse IgG (cat. no. 7076; 1:2000; Cell Signalling Technology) secondary antibody at room temperature for 1 h. To ensure equal loading of samples in each lane, the membranes were stripped and incubated with an anti‐*β*‐actin antibody (cat. no. MA1‐140; 1:2000, Thermo Fisher Scientific, Inc., Waltham, MA, USA). Reactive bands were detected using enhanced chemiluminescence reagents (Applygen Technologies, Inc., Beijing, China). The relative densities of the protein bands were quantitatively determined using ImageJ software (National Institutes of Health, Bethesda, MD, USA).

##### Ethics Approval

The mouse experiments were approved by the Ethics Committee of Fudan University (Shanghai, China) and Jinan University (Zhou Guang, Dong Guang, China) and were conducted according to the standard guidelines for the care of animals, which were approved by the Welfare Committee of the Center for Experimental Animals (Jinan University, Guangzhou, Guangdong, China).

##### Statistical Analysis

The statistical analysis was performed using SPSS version 22.0 software (SPSS, Inc., Chicago, IL, USA). All the data were analyzed using one‐way analysis of variance followed by least significant difference post hoc analysis or using an unpaired two‐tailed Student's *t*‐test (as appropriate). Statistically significant differences were denoted by *, *p* < 0.05; **, *p* < 0.01; and ***, *p* < 0.001.

## Conflict of Interest

The authors declare no conflict of interest.

## Author Contributions

Q.L., Z.X., M.Q., and I.S. contributed equally to this work. Q.L., Z.J.X., M.Q., Y.L.Y., S. X., and I.S. performed most of the experiments, analyzed data and wrote the manuscript. H.Z., L.P.L., J.S.K. and Y.H.C. reviewed and edited the manuscript. Q.H.Y., D.W., S.Y.C., T.J.F., D.A., X.H.Y., H.W., Z.Z.W., C.Y.W., H.Z.W., Y.H.Z., H.S.K., Q.S.Z., Q.T.Y., and Z.C. performed some experiments. J.S.K. and Y.H.C. provided valuable comments and reagents, analyzed the data and edited the manuscript. H.Z. and L.P.L. are the guarantors of this work and had full access to all of the data in the study, and takes responsibility for the integrity of the data and the accuracy of the data analysis.

## Supporting information

Supporting Information

## Data Availability

All the data are available within the article and Supplementary Information file, or available from the corresponding authors upon reasonable request.
