# Supplementary material for: Prodrug‐Loaded Zirconium Carbide Nanosheets as a Novel Biophotonic Nanoplatform for Effective Treatment of Cancer
Source: Adv Sci (Weinh). 2020 Nov 5;7(24):2001191. doi: 10.1002/advs.202001191 (PMC7740089; doi:10.1002/advs.202001191)
Supplement: Supplementary file 1 — Supporting Information [file ADVS-7-2001191-s001.pdf]

© 2020 Wiley-VCH GmbH

ADVANCED SCIENCE
Open Access

Supporting Information

for *Adv. Sci.,* DOI: 10.1002/advs.202001191

Prodrug-Loaded Zirconium Carbide Nanosheets as a Novel Bio-Photonic Nanoplatform for Effective Treatment of Cancer

Quan Liu, Zhongjian Xie, Meng Qiu, Inseob Shim, Yunlong Yang, Sisi Xie, Qinhe Yang, Dou Wang, Shiyou Chen, Taojian Fan, Bo Ding, Ziheng Guo, Dickson Adah, Xinhuang Yao, Yuhua Zhang, Hong Wu, Zongze Wu, Chaoying Wei, Hongzhong Wang, Hyeong Seok Kim, Qingshuang Zou, Qiaoting Yan, Zhen Cai, Jong Seung Kim, Li-Ping Liu,* Han Zhang,* and Yihai Cao**

Prodrug-loaded zirconium carbide nanosheets as a novel bio-photonic nanoplatform for effective treatment of cancer

Quan Liu[1,7,]†, Zhongjian Xie[2,]†, Meng Qiu[2,12,]†, Inseob Shim[3,]†, Yunlong Yang[4,5], Sisi Xie[5], Qinhe Yang[6], Dou Wang[1,7], Shiyou Chen[2], Taojian Fan[2], Bo Ding[13], Ziheng Guo[14], Dickson Adah[8,9], Xinhuang Yao[1], Yuhua Zhang[1], Hong Wu[1], Zongze Wu[1], Chaoying Wei[1], Hongzhong Wang[1], Hyeong Seok Kim[3], Qingshuang Zou[1], Qiaoting Yan[1], Zhen Cai[11], Jong Seung Kim[3,]*, Li-Ping Liu[1,10,]*, Han Zhang[2,]*, Yihai Cao[4,]*

Supplementary Materials

Figures

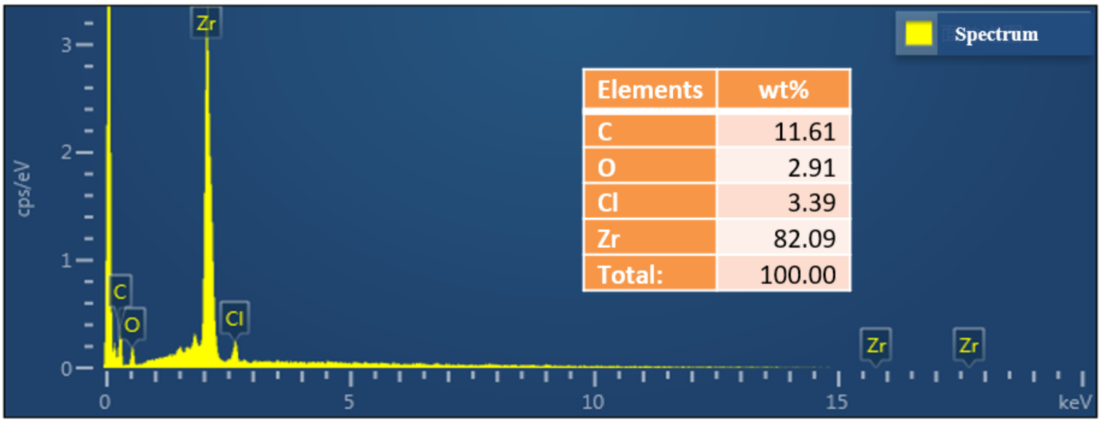

Figure. S1. EDS spectrum of ZrC NSs. The stoichiometric formula of ZrC is $Zr_{0.93}C_{1}O_{0.19}$.

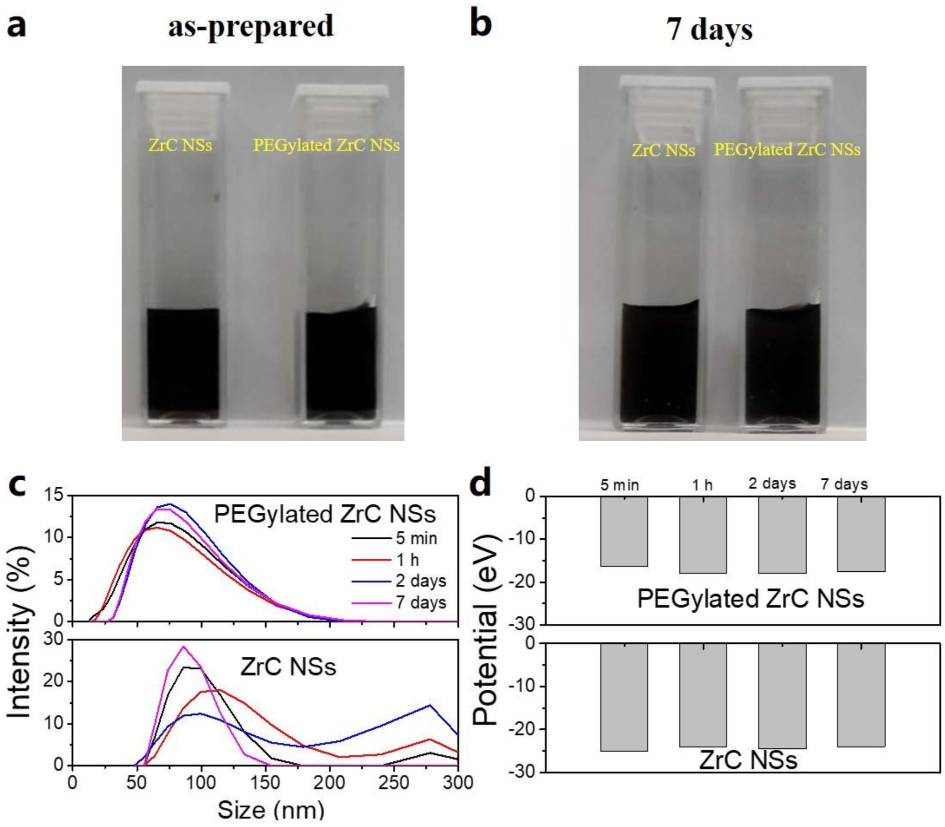

Figure. S2. Photographs of (**a**) ZrC NSs and PEGylated ZrC NSs in PBS solution, (**b**) ZrC NSs and PEGylated ZrC NSs in PBS solution after one week's rest. (**c**) DLS and (**d**) zeta potential of PEGylated ZrC NSs and ZrC NSs in PBS solution as time changed.

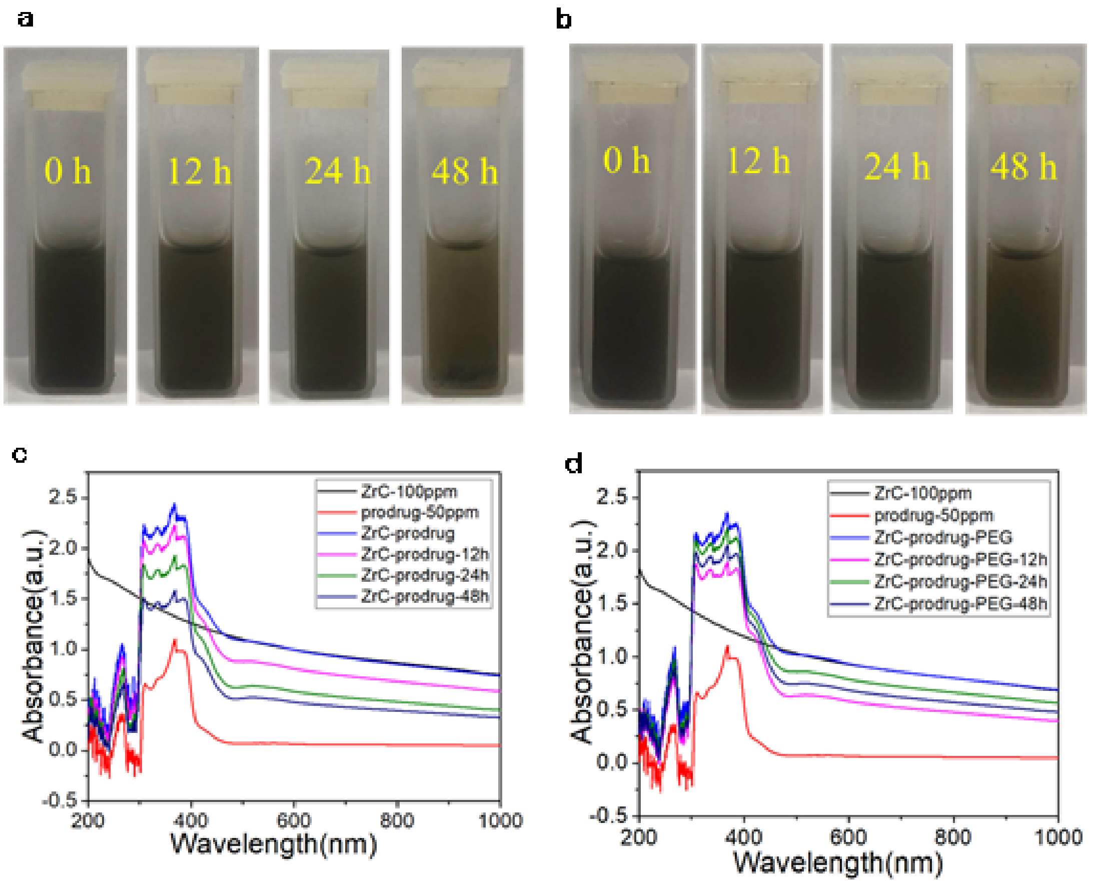

Figure. S3. The stability of ZrC@prodrug and ZrC@prodrug-PEG in serum. (**a**) Photograph of ZrC@prodrug in serum. (**b**) Photograph of ZrC@prodrug-PEG in serum. (**c**) The evolved absorbance of ZrC@prodrug for different time points. (**d**) The evolved absorbance of ZrC@prodrug-PEG for different time points.

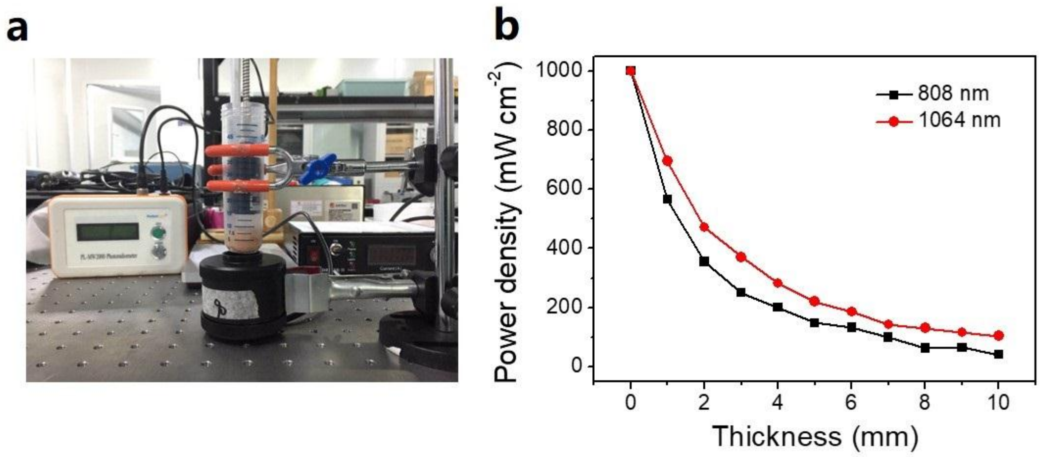

Figure. S4. (**a**) Equipment for detecting tissue penetration ability of NIR light at 808 and 1064 nm. (**b**) Power density of NIR-I (808 nm) and NIR-II (1064 nm) lights penetrating through tissues of different thickness intervals.

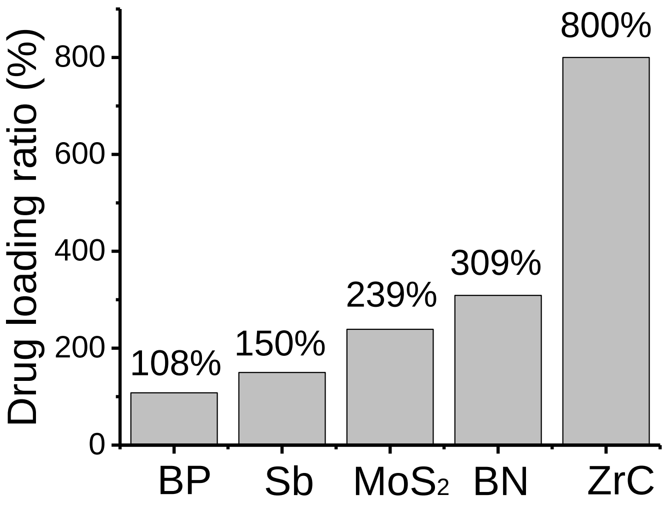

Figure. S5. Comparison of drug loading capacity of typical 2D materials.

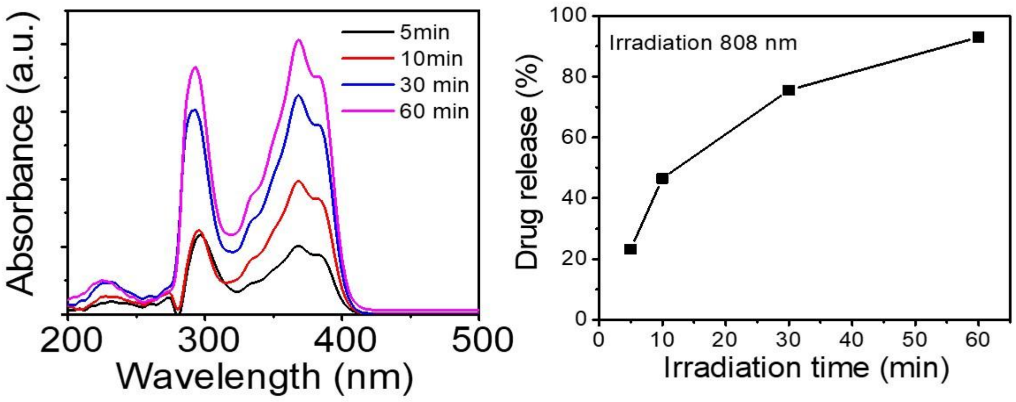

Figure. S6. Absorption spectra changes and drug release profiles with 808 nm laser irradiation.

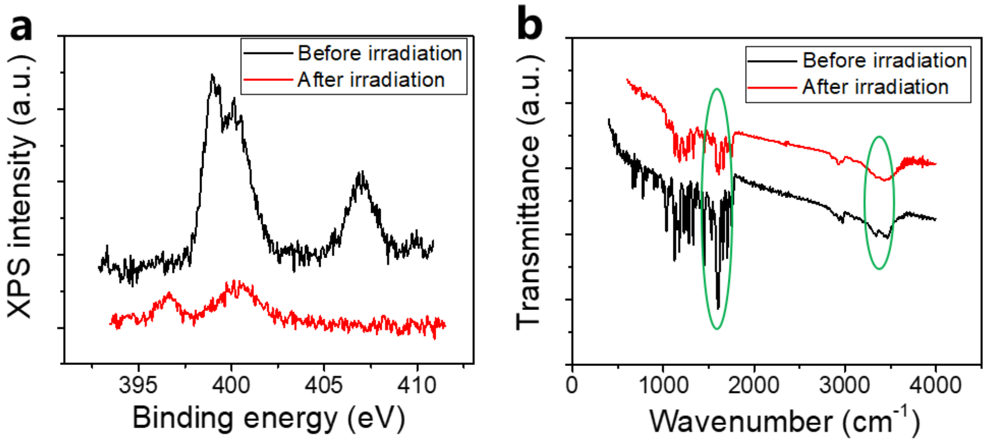

Figure. S7. (**a**) XPS and (**b**) FTIR spectra of prodrug-loaded ZrC NSs before and after irradiation.

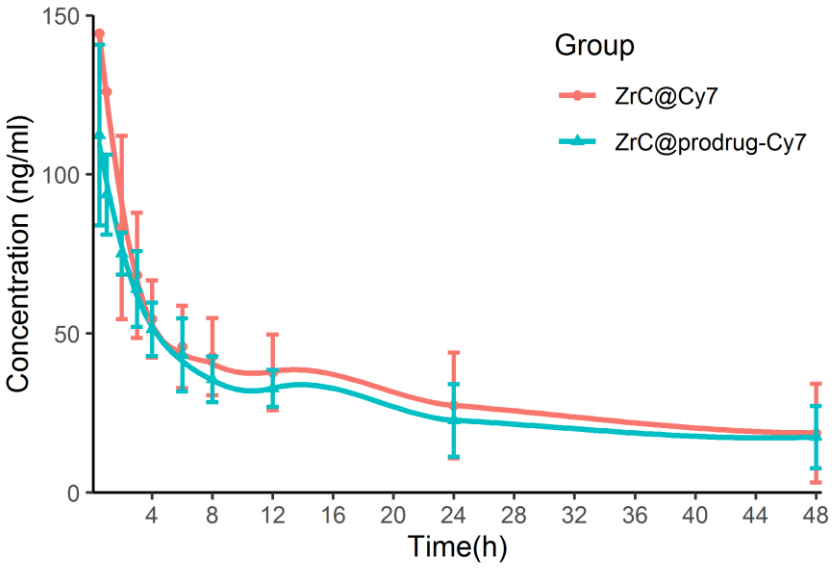

Figure. S8. Blood circulation curve of the ZrC@Cy7 and ZrC@prodrug-Cy7 determined by measuring the Cy7 fluorescence intensity in the blood of the Balb/c mice at different time points post-injection.

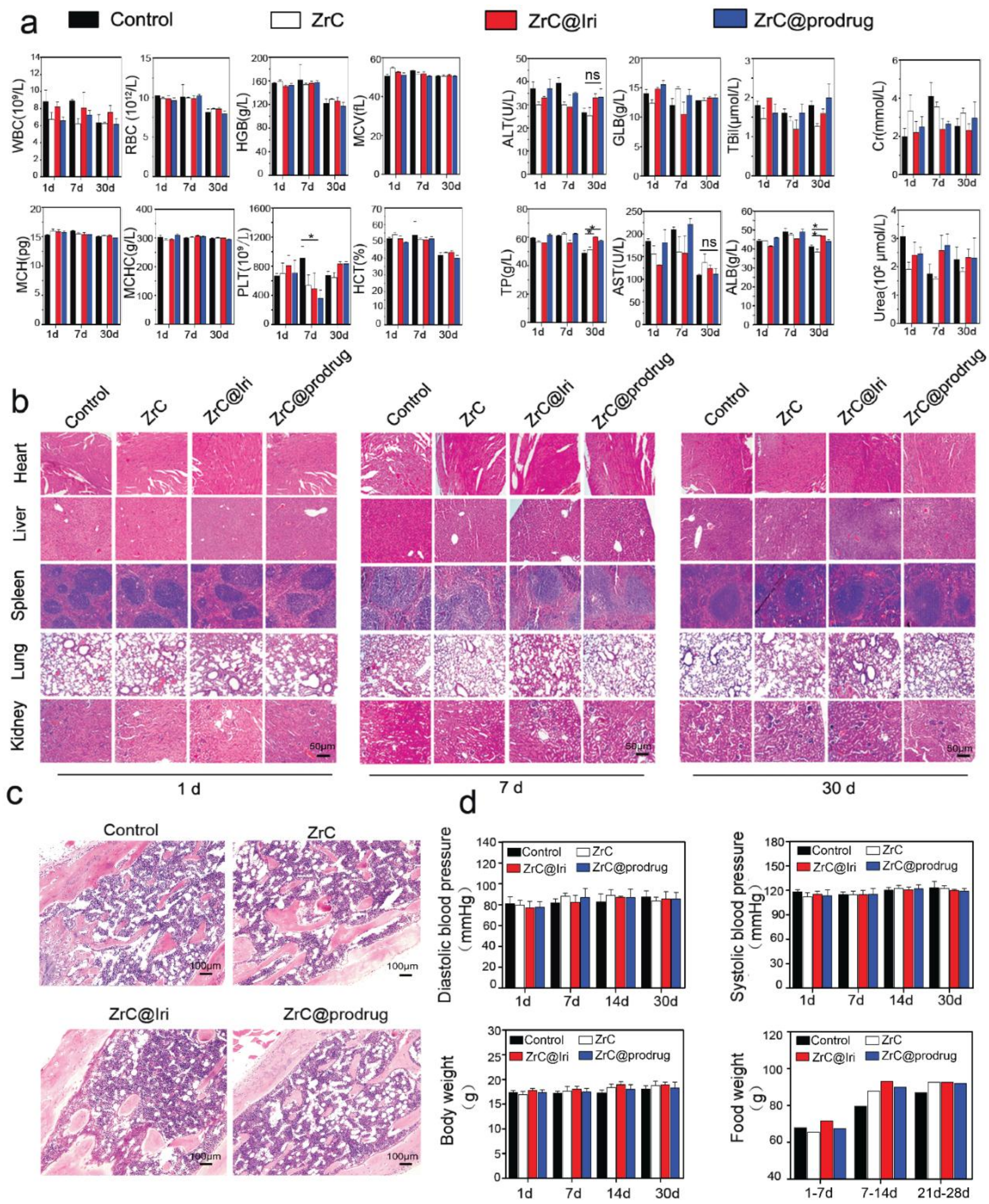

Figure. S9. Biocompatibility and cytotoxicity of ZrC nanoplatform with prodrug *in vivo*. (**a**) Measurement of haematological parameters, blood and urine biochemical indexes on the mouse groups intravenously injected with saline (control), PEGylated ZrC (ZrC), ZrC@Iri and ZrC@prodrug individually (10 mg kg^{-1}, 100 μL) at 1, 7- and 30-days post-injection. Data include indexes of white blood cells (WBC), red blood cells (RBC), haemoglobin (HGB), mean corpuscular volume (MCV), mean corpuscular haemoglobin (MCH), mean corpuscular haemoglobin concentration (MCHC), platelets (PLT), haematocrit (HCT), alanine transaminase (ALT), globulin

(GLB), bilirubin (TBIL), total protein (TP), aspartate transaminase (AST), albumin (ALB), urine creatinine (Cr) and urea. (**b**) Histological data (H&E) obtained from the liver, spleen, kidney, heart and lung of different treated mouse groups at 1-, 7- and 30-days post-injection. (**c**). H&E staining of bone marrow of the different treated mouse groups at 30-days post-injection. (**d**) Diastolic and systolic blood pressure, mouse body weight and food intake of the different treated mouse groups at 1-, 7- and 30-days post-injection. Bar graphs show the mean ± SD. *p<0.05, **p<0.01 and ***p<0.001. ns: no significance.

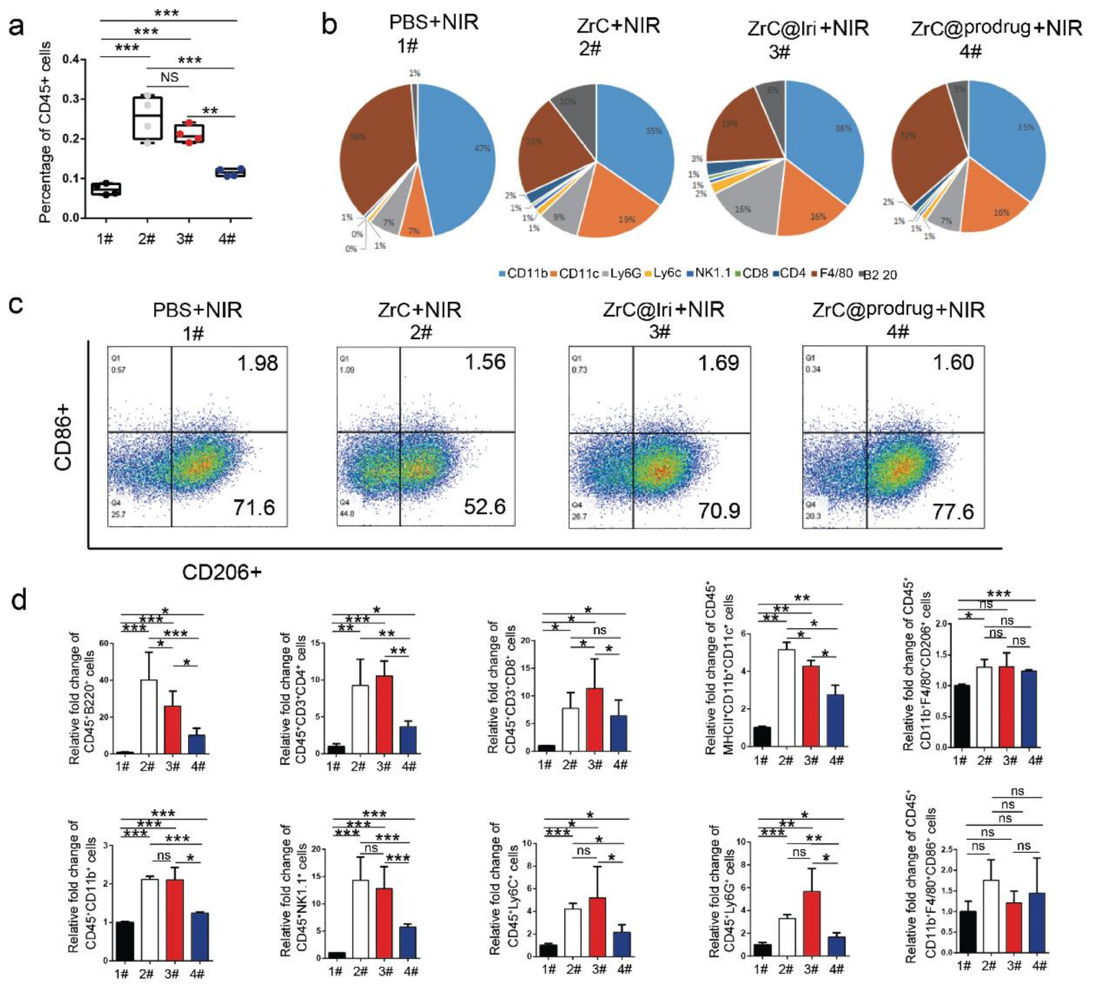

Fig.S10. Synergistic inhibition of tumor growth with decreased infiltration of inflammatory cells in visceral CRC models. (**a**) Quantification of $CD45^+$ cells in CRC tumors after treatments (control+NIR, PEGylated ZrC+NIR, ZrC@Iri+NIR, and ZrC@prodrug+NIR). (**b**) Pie charts of percentage of various inflammatory cells in CRC tumors after treatments. (**c**) Percentage of $CD86^+$ and $CD206^+$ macrophages in

the tumor microenvironment. (**d**) Quantification of CD45$^+$, B220$^+$ population; CD45$^+$, CD3$^+$, CD4$^+$ population; CD45$^+$, CD3$^+$, CD8$^+$ population; CD45$^+$, MHCII$^+$, CD11b$^+$ population; CD45$^+$, MHCII$^+$, CD11b$^+$, CD11c$^+$ population; CD45$^+$, NK1.1$^+$ population; CD45$^+$, Ly6C$^+$ population; CD45$^+$, Ly6G$^+$ population; CD45$^+$, CD11b$^+$, F4/80$^+$, CD86$^+$ population; and CD45$^+$, CD11b$^+$, F4/80$^+$, CD206$^+$ population in the tumor microenvironment after treatments. NIR: near-infrared radiation. Bar graphs show the mean ± SD. *p<0.05, **p<0.01 and ***p<0.001. ns: no significance.

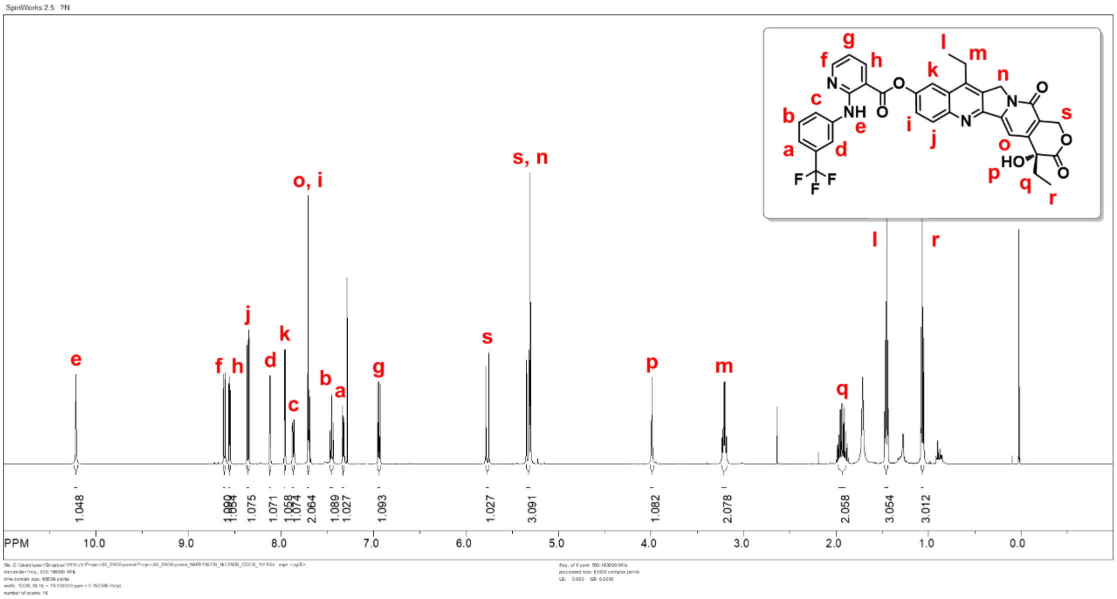

Figure. S11. ^{1}H NMR spectra (100 MHz) of prodrug Nif-SN38 in $CDCl_3$.

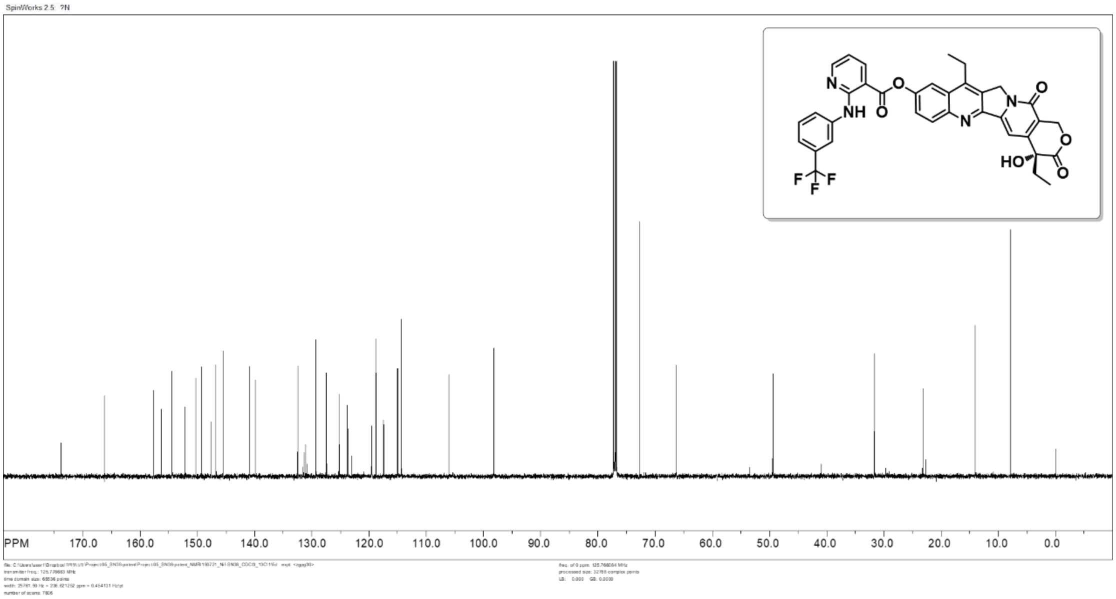

Figure. S12. ^{13}C NMR spectra (400 MHz) of prodrug Nif-SN38 in $CDCl_3$.
